# Dietary diversity and fish consumption of mothers and their children in fisher households in Komodo District, eastern Indonesia

**DOI:** 10.1371/journal.pone.0230777

**Published:** 2020-04-01

**Authors:** Emily Gibson, Natasha Stacey, Terry C. H. Sunderland, Dedi S. Adhuri

**Affiliations:** 1 Research Institute for the Environment and Livelihoods, Charles Darwin University, Casuarina, Northern Territory, Australia; 2 Department of Forest and Conservation Sciences, University of British Colombia, Vancouver, Canada; 3 Research Centre for Society and Culture, Indonesian Institute of Sciences, Jakarta, Indonesia; Emory University School of Public Health, UNITED STATES

## Abstract

Small-scale coastal fisheries contribute directly and indirectly to the food and nutrition security of marine-dependent households. Fishers can apportion part of their catch for household consumption or use the income earned to purchase staples and other desired foods. Fish are an important animal-source food rich in micronutrients essential for cognitive development of children and for adult health, and a valuable addition to rice-based diets. Furthermore, the engagement of women in fisheries value chains and increased control over income may facilitate decision-making which improves nutrition outcomes for women and their children. Despite these contributions, food insecurity remains prevalent in many low and middle income fish-producing countries. This paper reports findings from an exploration of the interplaying factors leading to food and nutrition insecurity in three marine-dependent coastal communities in eastern Indonesia, focusing on the consumption pathway, that is, the contribution of fish to the diets and nutrition of women and children. The research was undertaken as a mixed-methods case study. The study found that over 50% of mother-child pairs failed to meet the minimum recommended dietary diversity, and, while fish was the main animal-source food in diets, the introduction of fish to infant and young child diets was delayed due to fears of allergies and illnesses. Moreover, access to nutrient-dense foods was affected by variable and insufficient income from fisheries-based livelihoods, isolation from markets, and the broader food environment. Given the shift towards ‘nutrition-sensitive interventions’ to improve the livelihoods and well-being of fisher households, these results highlight the need for analysis of the intra-household sharing of fish within fisher households, culturally-appropriate strategies to improve the quality of family and especially complementary foods, and efforts to increase physical access to nutrient-dense foods.

## Introduction

There is growing recognition of the potential contribution of fish and small-scale coastal fisheries to food security and specifically in reducing micronutrient deficiency, especially for women and infants and young children [1–3]. Kawarazuka and Bene [4] categorise these contributions as occurring along three pathways. Firstly, an income pathway: fishers secure fish directly for household consumption or indirectly with the income earned through participation along the fisheries value chain used for the purchase of lower-value fish and other desired foods [5]. Worldwide, an estimated 36 million people are estimated to work directly in small-scale capture fisheries [6], and fishers may diversify their fishing effort and target species to maximise their catch and income. Fisheries thus have a dual role as both ‘food crop’ and ‘cash crop’ for households [4].

Secondly, a consumption pathway: fish are an important animal-source food in the diets of millions of people, providing over a third of the world’s population with almost 20% of their average per capita intake of animal protein [7]. Consumption of fish is even higher among coastal Indigenous populations, where it may provide up to 90% of per capita animal protein [8, 9]. Fish are a rich source of essential amino acids (especially lysine and methionine), long-chain polyunsaturated fatty acids, micronutrients such as vitamins A, B12 and D, and minerals including calcium, phosphorous, iodine, zinc, bioavailable heme iron, and selenium [1, 10, 11]. Oily fish such as anchovy and sardines are particularly rich in vitamin A [12]. Fish are an especially important component of–and addition to–plant-based diets which are dominated by calorie-dense staples such as rice [4, 12] because they enhance the bioavailability of non-heme iron and zinc from other foods consumed in the same meal [13, 14].

Thirdly, a distribution pathway: women’s participation in small-scale coastal fisheries value chains is argued to lead to their empowerment, with greater control over income resulting in increased spending on food (both staples and non-staples such as fish and vegetables) and health care for children, and thus improved nutrition outcomes [4, 15]. Consumption of a nutritious diet, inclusive of nutrient-dense animal-source foods such as fish is especially important for women of reproductive age (between 15 and 49 years of age) and infants and young children (six months to five years of age). Women have higher nutritional needs due to the demands of pregnancy and lactation, with research highlighting the importance of pre-conception health and nutrition [16]. For children, a nutritious diet in the first 1,000 days (from conception to their second birthday), and particularly from six months of age when breastmilk ceases to provide adequate nutrition and complementary foods are introduced, contributes to crucial stages of foetal neurodevelopment and child growth [17]. Poor foetal growth or stunting during this period are associated with short stature, lower school attainment, reduced adult income, and decreased offspring birthweight, leading to intergenerational consequences and significant economic and human capital costs [18, 19].

However, food insecurity, as evidenced by undernutrition, remains prevalent in low and middle income fish-producing countries despite the relative accessibility of fish and the economic opportunities offered through participation in small-scale coastal fisheries [4, 12, 20]. This is despite efforts over the last two decades to improve food security by increasing the availability of fish through improved management of fisheries and the use of landscape-scale approaches such as the ‘ecosystem approach to fisheries management’ and ‘marine protected area’ networks [21–23]. To date the evidence that such management approaches improve food security is mixed. For example, Darling [24] found that marine protected areas did not influence household food security (measured by protein consumption, diet diversity and food coping strategies) in coastal fishing communities in Kenya. On the other hand, Alva, Kiersten et al. [25] found that living in close proximity (< two km) to a marine protected area in the Philippines was positively correlated with children’s dietary diversity and intake of fish. Rather, the literature highlights that access, or one’s entitlement, to food–rather than the aggregate availability of food per se–is crucial and yet is determined by a complex interplay of institutional, social, cultural and economic factors, and the broader health environment (including access to maternal and child health services, and water, sanitation and hygiene) [5, 26]. This interplay is mapped in UNICEF’s ‘causes of malnutrition’ framework [4, and enhanced by: 15, 27]. Gender, and other intersectional factors (e.g. age, class, caste, among others), also affect access to food [15, 22, 28], and together with intra-household inequalities in access to and distribution of food, are often overlooked [21, 26].

This paper draws from research which investigates the contribution of fish and small-scale coastal fisheries livelihood activities to food and nutrition security, guided by Kawarazuka and Bene [4]’s pathways framework. The research was conducted in three island communities in Nusa Tenggara Timur (NTT), eastern Indonesia, where fisheries-based livelihoods are dominant and fish (marine fish and other edible marine resources (e.g. sea urchin, squid), hereafter ‘fish’)) was assumed to be readily available. Focusing on the consumption pathway, we (i) use food consumption and anthropometry data to assess the quality of diets of mothers and children; (ii) explore the importance of fish in diets and how fish and other nutrient-dense food groups are distributed within the household; and (iii) how gender (norms, roles and decision-making) affect household food and nutrition security. The study contributes to the literature by investigating intra-household distribution of nutrient-dense foods, namely fish, and the role of gender in access to and utilisation of food. In doing so it supports the development of improved nutrition-sensitive policy and program responses to undernutrition in Indonesia and in similar settings across the Indo-Pacific Region.

## Methods

### Ethics statement

This study was approved by the Human Research Ethics Committee at Charles Darwin University (H17085). The Indonesian Ministry of Research, Technology and Higher Education provided research clearance (247/SIP/FRP/E5/Dit.KI/IX/2017) and additional permission to conduct the research was obtained from NTT Provincial and Regency officials and village leaders. All respondents provided written informed consent to participate in the study, which was recorded on survey questionnaires or interview and focus group discussion participant forms. The names of the case study villages are not disclosed in this paper to maintain community confidentiality.

### Study setting

Indonesia, an archipelago of over 17,000 islands stretching 5,000 kilometres laterally between the Indian and Pacific Oceans [29], is the world’s second largest producer of marine fish in the world [30]. Much of these fish–up to 95%—are caught by small-scale fishers [31]: fishers who operate at the household level fishing with or without a fishing boat of small gross tonnage (typically <5 GT) and using unmechanized fishing gear [32]. It has been estimated that 2.6 million people are engaged in small-scale capture fisheries in Indonesia [33]; however given the informal and subsistence nature of fisheries activities across the archipelago and a tendency for official data to exclude or underestimate participation by women [34], the level of participation and catch is likely far higher. Nevertheless, fish are estimated to provide up to 54% of animal-source proteins [31]. Fish consumption varies widely depending on household livelihood strategy, economic position, geography (rural/urban) and cultural food preferences [35], with estimates of household fish consumption ranging from 4 kg/capita/year in urban Yogyakarta to 26.9 kg/capita/year in the eastern province of Maluku in 2011 [36]. More recently, nationally, consumption of fish was reported as 46.49 kg/capita/year in 2017, with the government hoping to increase this to 50.65 kg/capita/year in 2018 [37].

Marine capture fisheries production in Indonesia is however threatened by destructive and unsustainable fisheries practices, land-based development (leading to pollution), as well as potential damage and species disruptions caused by climate change [29]. It is estimated that the majority of targeted fish stocks are fully exploited or overexploited, leaving little opportunities for increased production [38]. Indonesia was instrumental in establishing the Coral Triangle Initiative on Coral Reefs, Fisheries and Food Security (CTI-FFS) in 2009, aiming to improve management of biodiverse coral reef ecosystems found in member States, while simultaneously increasing food security [39]. In doing so, Indonesia committed to protecting 20 million hectares of its marine and coastal area by 2020. However, Foale, Adhuri et al. [22] point out that there had yet to be a clear articulation of how the CTI activities would result in improved food security. A decade later, Indonesia has made limited progress in meeting global nutrition targets, with 36.4% of children aged under five years having stunted growth and just over 20% of adults overweight [7, 17].

The area selected for this case study was Komodo District, West Manggarai Regency, NTT. Livelihoods across the Province are centred around rain-fed agriculture or, along the coast, fisheries. In 2015, the Province had the third highest rate of household poverty in Indonesia, with 22.61% of households living below the national poverty line, and many Regencies experienced high levels of food insecurity, as evidenced by a very high rate (51.73%) of stunting among children under five years of age [40]. Previous food security assessments focused on farming communities have identified a combination of factors contributing to high levels of food insecurity and undernutrition along the access and utilisation dimensions of food security: poor dietary intake, improper child feeding practices, and poor water, sanitation and hygiene conditions [41, 42].

The three island communities purposively selected were situated on islands 7–10 kilometres from Labuan Bajo, the Regency and District capital, in the Sape Straits. The communities comprised households identifying as of Bajau or Bugis ethnicity, engaged in various small-scale fisheries livelihood activities including capture fisheries (demersal, pelagic and reef fish), collection and trade (for local and export markets), and post-harvest processing (drying). The area has been characterised by rapid development linked with visitation to Komodo National Park which was established in 1980. A management plan enacted in 2000 delineates zones of use for terrestrial and maritime areas and regulates the type and methods of fishing [43], and, subject to these restrictions, fishers from the case study communities are permitted to fish within the Park’s boundaries.

Each of the communities is served by a basic health post and more serious illnesses are referred to the health centre or private hospital in Labuan Bajo. Basic maternal and child health services, including antenatal checks and immunisations, are delivered in each of the communities.

Perishable (fish, eggs, fruits and vegetables), staple (rice) and packaged foods are available in each of the communities through small vendors. These vendors purchase perishable foods (fruit and vegetables, eggs) from wholesalers or large markets in Sape (on neighbouring Sumbawa Island) typically on a weekly basis and transport it to the communities via inter-island and local boat services. There is no cultivation, and the small livestock (chickens, ducks and goats) present are reserved for ceremonial feasts or sold for supplementary income.

### Field methods

The research applied mixed methods, with fieldwork conducted between October 2017 and May 2018. The methods included a household survey, focus groups discussions, semi-structured interviews, and a market survey.

#### Research team

The research activities were conducted by the first author (the researcher), a post-graduate student from Australia, and three field assistants. The field assistants were recent female university graduates in fisheries or public health fields; two were from Kupang on Timor Island while one was from Labuan Bajo and had a familial-social network across each of the communities. The field assistants were selected based on their knowledge and experience, willingness to travel, familiarity with life in coastal communities and ability to communicate and develop rapport with community members. The field assistants received training on key elements of the research framework, survey administration, interviewing techniques and facilitating discussion groups. Interview/discussion guides were developed by the researcher, in English, and reviewed with the field assistants to ensure understanding and accurate translation of key concepts, and implemented in Indonesian or the local language, in which case one field assistant acted as a secondary translator.

Data collection was facilitated by the researcher and field assistants staying in two of the communities and in close proximity to the third and smallest community during the fieldwork periods. Fieldwork periods were planned based on the identification of dry and wet seasons and to avoid the Islamic holy month of Ramadan, as food consumption patterns and participation in livelihood activities were reported to be different during this month. An initial scoping trip of 8 days was undertaken in March 2017. A total of 93 days were spent in the field between October 2017 and May 2018. This enabled the researcher to develop familiarity and rapport with community members and to observe daily activities such as the early morning landing and subsequent processing of fish, food preparation activities, preparations for celebrations such as weddings, and for informal conversations between the researcher, field assistants, fishers and families. The researcher made field notes of observations, experiences and thoughts during the fieldwork periods.

#### Household survey

Sixty-six households participated in the study (Table 1). The sample size was determined based on the estimated prevalence of child stunting for Komodo District (49.31%) [40] and estimated number of eligible households (that is, those comprising a woman of reproductive age (hereafter referred to as mothers or women) and a child aged between six months and five years (hereafter referred to as child/children or by the relevant age category)) [44]. A list of eligible households was prepared with the assistance of local health staff and, as Indonesian villages are administratively divided into sections reflecting settlement and expansion of the village, five randomly selected eligible households from each section were surveyed. The questionnaire was pre-tested in a similar mainland community.

**Table 1:** Summary of field methods and data collection.

Research activities	Field site				Total
	FS 1	FS 2	FS 3	Labuan Bajo	
Population / households ^a^	1,860 / 477	604 / 152	242 / 56		2,706 / 685
Eligible households ^b^	186	52	25		263
Household survey					
• January 2018	36	20	10		66
• April 2018	34	15	10		59 ^c^
Interviews					
• Village leaders and government reps	5 (3M; 2W)	3 (2M; 1W)	2 (1M; 1W)	6 (2M; 4W)	16 (8M; 8W)
• Community member	12 (0M; 12W)	6 (0M; 6W)	4 (0M; 4W)	2 (0M; 2W)	24 (0M; 24W)
Focus group discussions	5 (8M; 29W)	2 (4M; 10W)	2 (6M; 5W)		9 (18M; 44W)

FS–field site; M–man; W–woman

^a b^ estimates from village census information and local health volunteers respectively, October 2017

^c^ two households declined to participate again and the other households were absent.

The questionnaire collected information on the following characteristics: (a) mother’s age, education, consumption of food and beverages using the 24-hour recall method, livelihood activities, participation in gleaning and household decision-making, and health and nutrition knowledge; (b) child’s age and sex, feeding practices (including breastfeeding, age at introduction of complementary foods, consumption of food and beverages using the 24-hour recall method; and (c) characteristics of the household (house construction and access to improved water, sanitation and electricity), ownership of durable and livelihood assets, ownership of livestock, and production of food crops (see S1 Data and S2 Data). Data on mother and child consumption of food and beverages was collected twice, once during the wet season and once during the dry season. Repetition allowed for the capture of seasonal variations in access to, availability of and consumption of foods. Anthropometry data for women and children was also collected during the first survey period.

#### Interviews and focus group discussions

Semi-structured interviews and focus group discussions were conducted between October 2017 and May 2018 (Table 1). Topics covered included: (1) identification of seasons and associated change or differences in livelihood activities, food and water availability, and health and nutrition issues; (2) historical and contemporary food consumption and meal patterns; issues with food access including taboos (proscriptions) or intra-household distribution practices; child feeding practices; perceptions and understanding of healthy foods; and (3) women’s roles and responsibilities within the household and at the community level.

Interviews were conducted with key informants (village leaders and local health staff as well as government representatives from relevant agencies) and with female community members, until no new information arose. Interviews were conducted in the respondent’s office or home, and lasted for between 30 and 75 minutes. Participants for focus group discussions were initially identified by key informants based on perceived knowledge or experience, with snowball sampling used for subsequent discussion groups relating to women’s roles, child feeding practices, and health and nutrition knowledge. Initial discussion groups (one in each community) comprised both males and females, however remaining discussion groups comprised women only. Focus group discussions were conducted in community meeting places and ran for approximately 45 minutes.

Interviews were recorded, unless the participant declined (n = 2), but focus group discussions could not be recorded due to surrounding noise. In these instances, detailed notes were recorded as the interview or discussion progressed. The research team reviewed the key themes, points and anecdotes immediately or shortly after each interview and discussion group, with the researcher recording additional notes in English and verifying these with the field assistants. Interview recordings were subsequently transcribed and translated by the field assistants.

#### Data analysis

*Maternal and child dietary diversity*. Data on mother and child food and beverage consumption was collected using the modules developed by FAO and FHI 360 [45] and WHO [46]. The modules were adapted based on information about contemporary dietary patterns collected in interviews and focus groups and a market survey of locally available foods. Additional questions were added about the consumption of fish (species and whether consumed fresh or dried), and other oils and fats, savoury and fried snack foods, sweet snacks and sugar-sweetened beverages. Fortified foods, infant formula and milk products, and all snack foods were classified in accordance with the relevant guidance manuals. A list method, in which the respondent was asked if she (or her child) had consumed foods from a particular food group in the preceding 24-hour period followed by prompting about different meals and snacks and examples of locally-available foods, was used. Consumption of at least 5 of 10 food groups for women and at least 4 of 7 food groups for children (the thresholds) is positively associated with micronutrient adequacy, an important indicator of dietary quality [45, 46]. The data were entered into Microsoft Excel spreadsheets and descriptive statistics for maternal and child food group consumption, comparisons within mother-child pairs, and the proportion of mothers and children having adequate dietary diversity are presented.

*Anthropometry*. Anthropometry (height and weight) for mothers and children were collected in accordance with standardised procedures [e.g. 47]. Data were entered into Microsoft Excel spreadsheets. Maternal body mass index (BMI) was calculated and grouped as underweight (<18.49), normal (18.5≤24.9), overweight (>25≤29.9) and obese (≥30) [48]. Child stunting (height-for-age, HAZ) and wasting (weight-for-height, WHZ) were calculated in WHO Anthro v.3.2.2, with children with HAZ and WHZ below -2 SD from the median of the reference population classified as stunted or wasted respectively [49].

*Household wealth*. Household wealth was evaluated by a Material Styles of Life scale based on the building materials of the house, access to electricity (none, purchased or own source), access to improved sanitation, and the presence or absence of household assets (fan, television, mobile phone, tablet, fridge, couch/sofa set, washing machine, scooter) [50, 51]. These items were factor analysed using the principal component method, and items with low factor loadings removed [52]. The first principal component axis explained 27.72% of the variation in wealth among households. The scale was weighted by the number of household members and households were grouped into three wealth levels: low (lowest 40%), middle, and high (highest 20%).

Additional analyses were undertaken in SPSS (version 24, IBM), using data for the 59 mother-child pairs for which there was data in both seasons. Differences between women’s and children’s dietary diversity scores and the proportion with adequate dietary diversity in wet and dry seasons were explored using Wilcoxon signed-rank and McNemar’s tests. Differences between women’s and children’s seasonal consumption of food groups were explored using McNemar’s test. Logistic generalised linear mixed models (binominal with logit link, with household as a random intercept and variance component as covariance structure) were tested to assess the association between a child having adequate dietary diversity and different socio-demographic factors. A multivariable model was developed with confounders (age) and variables with *p* < 0.1 from the univariable model included as predictors. Model diagnostics for the final model included a low Akaike Information Criterion and residual diagnostics, model convergence and absence of multicollinearity as indicated by variance inflation factors (VIF < 1.2). Two-tailed *p* values <0.05 were considered significant.

The interview and focus group discussion data, along with field notes, were entered into NVIVO Pro 12 (QSR) and analysed by the lead author using a combination of deductive and inductive coding based on the pathways framework. Where local terms are included, the Indonesian-English translation is provided at the first instance and the Indonesian word is italicised thereafter.

## Results

### Characteristics of women and children

The average age of the women was 29.7 years (SD +/- 6.7 years; range 19, 43) (Table 2). Nearly half of the women (48.5%) had not completed primary school, with only 22.7% attempting or completing a higher level of education. Forty-four percent of the women sampled had a BMI within the normal range, while 46% of women were classed as overweight or obese.

**Table 2:** Socio-demographic characteristics and nutritional status of women and children.

Characteristics	Mean ± *SD* or *n* (%)		N
*Maternal characteristics*			
Maternal age, years	29.7 ± 6.7		66
Highest level of schooling completed			66
Some primary	32 (48.5)		
Completed primary	19 (28.8)		
Some or completed secondary; or further education	15 (22.7)		
Mother has income-generating livelihood activity	26 (39.4)		
Nutritional status			63 ^a^
Underweight (BMI < 18.49)	6 (9.5)		
Normal (BMI ≤ 18.5 < 24.9)	28 (44.5)		
Overweight (BMI ≤ 25 < 30)	23 (36.5)		
Obese (BMI ≥ 30)	6 (9.5)		
*Child characteristics*			
Sex			66
Male	41 (62.1)		
Age (wet / dry season)			66, 59
6–11 months	8 (12)	5 (8.5)	
12–17 months	14 (21)	10 (16.9)	
18–23 months	8 (12)	7 (11.9)	
24+ months	36 (55)	37 (62.7)	
Breastfeeding (wet / dry season), by age category ^b^			66
6–11 months	8 (100)	4 (80)	
12–17 months	11 (79)	8 (80)	
18–23 months	6 (75)	4 (57)	
24+ months	15 (41.7)	14 (38)	
Minimum acceptable feeds (wet / dry season), by age category ^b^ ^c^			66, 59
6–11 months	7 (87.5)	2 (40)	
12–17 months	4 (28.6)	5 (50)	
18–23 months	3 (37.5)	2 (28.6)	
24+ months	19 (52.8)	19 (51.4)	
All children	33 (50)	28 (47.5)	
Stunting, by age category ^b^			56 ^d^
6–11 months	3 (37.5)		
12–17 months	4 (33.3)		
18–23 months	2 (33.3)		
24+ months	19 (63.3)		
All children	28 (50)		
*Household*			
Material Styles of Life scale (wealth)	0, ± 1 Range -1.21805, 2.24643		66
Lowest wealth	24 (36.4)		
Middle wealth	30 (45.5)		
Highest wealth	12 (18.2)		
Access to improved sanitation ^e^	25 (37.9)		66
Dual burden households ^f^	9 (16.98)		53

^a^ Height and weight of pregnant women was not recorded

^b^ as a proportion of relevant age class

^c^ the minimum acceptable number of feeds per day for a breastfed child aged 6–8 months is 2, for a breastfed child aged 9–23 months is 3, and for a non-breastfed child aged 9–23 months is 4 feeds [46]

^d^ height and weight could not be collected from 10 children

^e^ households had access to improved sanitation if they had a pour/flush to latrine pit or septic tank that was not shared with other households

^f^ households were classed as ‘dual burden’ when the mother has a BMI class of overweight or obese and the child has stunted growth.

Just under two thirds (62.1%) of children included in the sample were male (Table 2). Over half of the children (55% in commencing wet season) were 24 months and older. The majority of the children had been breastfed, with only 13% of the children never breastfed or with breastfeeding ceasing at less than six months of age. Continued breastfeeding (with feeds continuing beyond two years of age) was widely practiced, with 27% of the children still breastfed aged more than two years. Semi-solid or solid foods were most often introduced to children aged between five and six months; however, these foods were introduced to one fifth of children when aged less than four months and delayed beyond 12 months for eight percent of children. Overall, fifty percent of children had stunted growth, with the proportion of stunting higher among children aged 24 months or more.

Households with higher wealth, as indicated by higher Material Styles of Life scores, had homes with brick walls and cement floors, their own source of electricity and access to improved sanitation. In contrast, households with lower wealth had homes with iron/tin sheet walls and timber floors, and purchased electricity from private vendors or a village system when possible.

The water, sanitation and hygiene environment was challenging in all of the communities. Two of the communities lacked a fresh water source, and the third had access to an unprotected spring, with reduced flow towards the end of the dry season. Most households relied on the delivery of bulk water from the mainland, and on bottled water, however water storage equipment and containers were not routinely maintained or cleaned. Only 37.9% of households had access to improved sanitation, with many households reliant on shared toilet facilities or resorting to open defecation.

### Dietary diversity of women and children

**Fig 1:**
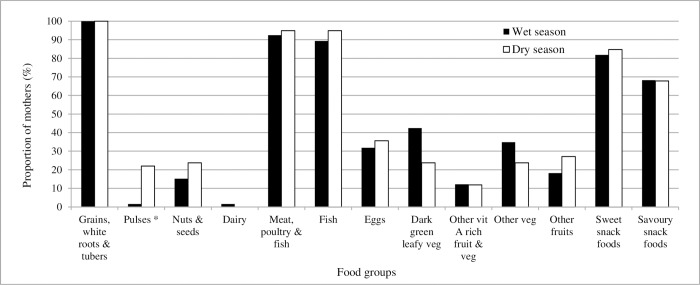
Proportion of mothers consuming different food groups during the wet and dry seasons. Fig 1 shows the proportion of mothers who reported consuming foods within different food groups in the preceding 24-hour period. Consumption of fish is reported within the food group ‘meat, poultry and fish’ and as in individual food; consumption of sweet and savoury snack foods are not included in the dietary diversity indicator score, but are included to show dietary pattern. * indicates a statistically significant difference in seasonal consumption.

Maternal dietary diversity was low: less than one quarter of mothers consumed foods from five or more of the 10 food groups in either recall period (21.2% wet season and 23.7% dry season) (Table 3). In the wet season the average number of food groups consumed was 3.5 (median 3; range 1–7). Two-fifths (40%) of mothers consumed foods from at least three food groups: ‘grains, white roots and tubers’ (rice), ‘meat, poultry and fish’ (fish), and ‘dark green leafy vegetables’ (water spinach) (Fig 1). In the dry season, the average number of food groups consumed was 3.6 (median 4; range 1–7). Just under one third (32%) of mothers consumed foods from at least three food groups: ‘grains, white roots and tubers’ (rice), ‘meat, poultry and fish’ (fish), and ‘eggs’. There was a statistically significant seasonal difference only in mother’s consumption of pulses (*p* = 0.002). Consumption of low-nutrition density foods was high, with over 80% of women consuming sweet snack foods (e.g. home-made or packaged biscuits and cakes) and over 67% of women consuming savoury snack foods (e.g. fried bananas, fish balls) in both recall periods.

**Table 3:** Summary of mother and child dietary diversity scores and proportion of individuals and pairs achieving minimum recommended dietary diversity.

**A.** Mothers and children	Wet season		Dry season	
	Mean; median; range	Proportion (%) achieving threshold	Mean; median; range	Proportion (%) achieving threshold
Mothers	3.5; 3.0; 1–7	21.0	3.6; 4.0; 1–7	24.0
Children				
6–11 months	1.5; 1.0; 0–3	0.0	1.4; 1.0; 0–3	0.0
12–17 months	2.4; 2.5; 0–4	7.1	3.0; 3.5; 1–4	50.0
18–23 months	2.8; 3.0; 0–5	37.5	2.7; 2.0; 1–5	28.6
24+ months	3.2; 3.0; 1–6	33.3	3.4; 3.0; 1–6	48.6
All children	2.8; 3.0; 0–6	24.2	3.1; 3.0; 0–6	42.2
**B.** Mother-child pairs		Proportion (%) of pairs		
		Wet season		Dry season
Both mother or child met threshold		14		20
Only child met threshold		11		24
Only mother met threshold		8		3
Neither mother or child met threshold		68		53

The minimum recommended dietary diversity (“the threshold”) for children is 4 of 7 food groups, and for women of reproductive age is 5 of 10 food groups.

**Fig 2:**
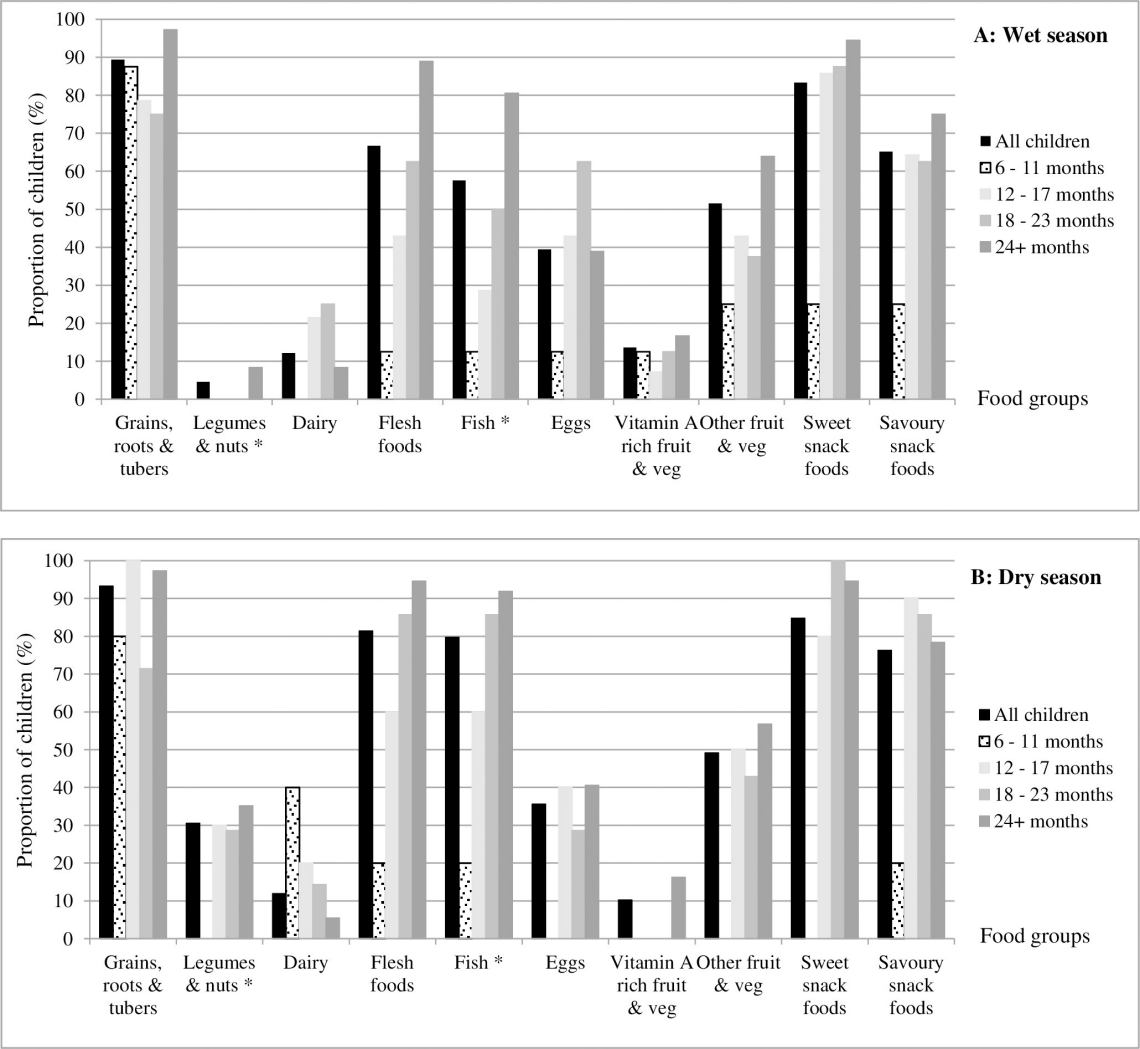
Proportion of children consuming different food groups during the wet and dry seasons. Panels A and B show the proportion of children, by age class, who were reported to have consumed foods within different food groups in the preceding 24 hour period in the wet and dry seasons respectively. Consumption of fish is reported within the food group ‘flesh foods’ and as an individual food; consumption of sweet and savoury snack foods are not included in the dietary diversity indicator score, but are included to show dietary pattern. * indicates a statistically significant difference in seasonal consumption for ‘all children’.

The dietary diversity of children was also low (Table 3). Less than one quarter (24%) of children consumed foods from four or more of the food groups in the wet season recall period; however, a higher proportion of children (42%) consumed foods from four or more of the food groups in the dry season recall period. None of the infants (6–11 months) met the dietary diversity threshold in either season. In the wet season, the average number of food groups consumed by children was 2.8 (median 3; range 0–6); dietary diversity increased with the age of the child. Overall, two-fifths (40.9%) of children consumed foods from at least three food groups (Fig 2): ‘grains, roots and tubers’ (rice), ‘flesh foods’ (fish) and ‘other fruits and vegetables’ (water spinach) over the course of the preceding 24 hours. In the dry season, the average number of food groups consumed by children was 3.1 (median 3; range 0–6) and similarly increased with the age of the child. Just under half of all children consumed foods from at least three food groups (Fig 2): ‘grains, white roots and tubers’ (rice), ‘flesh foods’ (fish), and ‘other fruits and vegetables’ (*kedondong* or ambarella). There were statistically significant differences in children’s (all age groups combined) seasonal consumption of legumes and nuts (*p* = 0.001), dark leafy green vegetables (*p* = 0.013), and fish (*p* = 0.004). Consumption of low-nutrition density foods was high among children, with around 90% of mothers reporting that their child had consumed sweet and/or savoury snack foods during the recall periods.

There were not statistically significant differences across seasons between mother and children’s median dietary diversity scores (mother: *z* = 0.065, *p* = 0.948; children: *z* = 1.669, *p* = 0.095), nor the proportion of women and children having adequate dietary diversity (mother: *p* = 1.000; children: *p* = 0.078).

**Fig 3:**
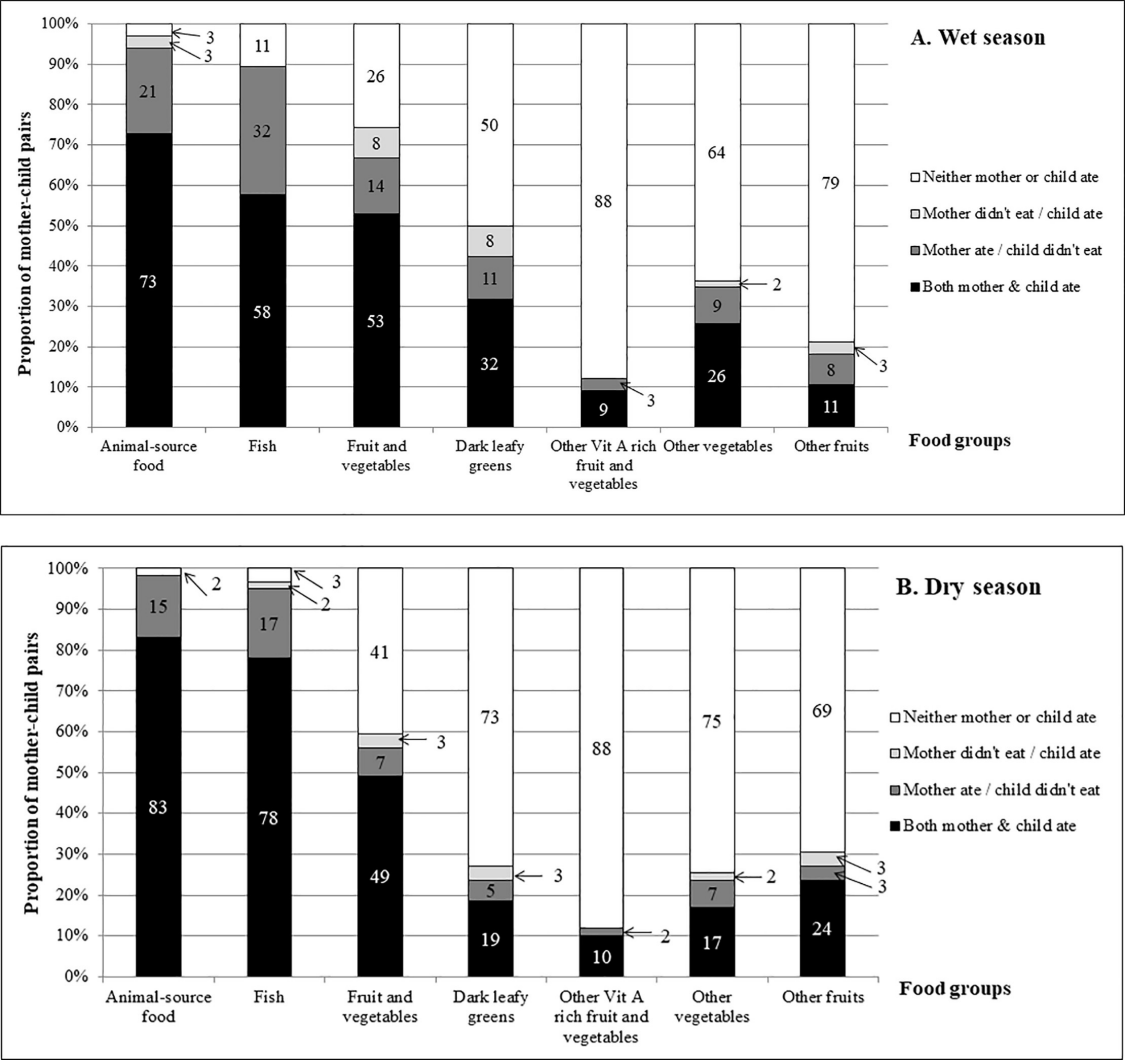
Proportion of mother-child pairs consuming nutrient-dense food groups in the wet and dry seasons. Fig 3 shows the proportion of mother-child pairs who consumed foods from different food groups during the wet and dry season recall periods. While the diets of mother-child pairs are similar, mothers reported consuming fish and ‘fruits and vegetables’ more often than their child.

When assessed as mother-child pairs, only 14% and 20% of mother-child pairs both had adequate dietary diversity in the wet and dry season respectively (Table 3). Conversely, neither mother nor child had adequate dietary diversity in the wet (68% pairs) and dry (53% pairs) season. While the diets of mothers and children were substantially similar, mothers reported consuming animal-source foods and ‘fruits and vegetables’ (all sub-groups combined) more frequently than their children (Fig 3).

### Consumption of fish

Fish was the most frequently consumed animal-source food by mothers, with around 90% of mothers consuming fish during the wet and dry season 24-hour recall periods (89.4% and 94.9% respectively). Fish was mostly consumed fresh (89.4% wet season and 91.5% dry season), with 39% and 27% of mothers consuming dried fish in the wet and dry seasons respectively. Fish was also the most frequently consumed animal-source food by children, with 57.6% of children consuming fish in the wet season and 79.7% consuming fish in the dry season. Consumption of fish was higher in the dry season among all age groups, and was substantially higher among younger children (12–17 months: 28.6% wet season, 60% dry season; 18–23 months 50% wet season, 85.7% dry season). However, consumption of fish was low among infants: only 12.5% and 20% of infants had consumed fish during the wet and dry season recall periods respectively. Over three quarters of infants had not received any animal-source food in either recall period. Increased consumption of fish by young children in the dry season data collection period was likely the result of a large harvest of small pelagic fish and squid at this time, described by fishers and women as “a flood of fish”, and with women assisting in the sorting and laying out of fish to dry receiving payment in fish. Children who did consume fish were more likely to have meals prepared from fresh fish than dried fish. In the initial univariable analysis, children who had consumed fish were five times more likely to have adequate dietary diversity (OR 5.0, CI 1.7–15.1, *p* = 0.005) (Table 4).

**Table 4:** Association between a child having adequate dietary diversity and explanatory factors.

Factors	*Univariable model*			*Multivariable model*		
	Odds ratio	95% Cl	*p* value	Odds ratio	95% CI	*p* value
Child’s sex (F/M)	0.5	0.2–1.3				
Child age (months)	1.0	1.0–1.1	0.034 *	1.1	1.0–1.1	0.032 *
Child’s consumption of fish (Y/N)	5.0	1.7–15.1	0.005 **			
Child breastfeeding (Y/N)	0.7	0.3–1.5				
Child stunted (Y/N)	2.6	0.7–9.3				
Maternal age (years)	1.0	1.0–1.1				
Mother’s dietary diversity achieved	9.9	3.8–25.9	< 0.001 ***	20.0	6.0–66.1	< 0.001 ***
Mother with completed primary or higher education	1.7	0.7–4.1				
Mother with own livelihood activity	1.7	0.7–4.1				
Not a dual burden household	0.3	0.1–0.6	0.009 **	0.4	0.1–1.3	0.106
Access to improved sanitation	1.0	0.4–2.3				
Season	2.3	1.2–4.6	0.018 *	2.9	1.1–7.9	0.035 *

CI: Confidence Interval; *p* value only shown when significant

* *p* < 0.05

** *p* < 0.01 and

*** *p* < 0.001.

### Intra-household distribution of nutrient-dense foods and food taboos

Intra-household differences in the consumption of nutrient-dense foods, especially fish, were driven by taboos about suitable foods for infants and young children, and pregnant and lactating women. The greatest disparity in mother-child diets occurred with respect to the consumption of fish: 32% of mothers consumed fish when their child did not in the wet season and 17% consumed fish when their child did not in the dry season (Fig 3). Mothers did not give infants and younger children fish or sea urchins because they were believed to cause allergies or stomach upsets. Breastfeeding women were proscribed from eating parrotfish and rabbitfish, in addition to sea urchins and shellfish, because they were said to cause upset stomachs in the breastfed child (women in FG FS1-2, 2017–18), while consumption of white snapper by the mother was believed to cause *a white growth in the infant’s mouth* (woman, FS1, 2017). Finally, pregnant women were proscribed from eating common and long-spined sea urchin for fear it would cause uterine bleeding.

The taboos were handed down from mother-to-mother, such that a woman who consumed proscribed foods was said to be *disobeying her mother* (women, FS3, 2018), although it was acknowledged as ultimately the woman’s choice. Mothers reported delaying the introduction of taboo species until the child had ceased breastfeeding, could eat un-mashed rice, or had started walking.

### Factors affecting the achievement of children’s dietary diversity

The association between a child having adequate dietary diversity and numerous independent variables was assessed: child characteristics (age, sex, consumption of fish, breastfeeding status, stunted), mother’s characteristics (maternal age, maternal level of schooling, maternal income-generating livelihood activity, mother having adequate dietary diversity), household characteristics (household wealth, access to improved sanitation), and season. The final model showed that the strongest predictor of a child having adequate dietary diversity was their mother having adequate dietary diversity (Table 4).

### Gender roles and food provisioning

Women were responsible for the acquisition of food, planning of meals and preparation of foods. Household roles were prescribed by local culture: *household matters–from taking care of children*, *of the house–it’s all the responsibility of the wife and*, *if there is a daughter*, *her daughter; its taboo for a man to do a woman’s job* (woman, FS1, 2017). Women were also responsible for managing household monies. One woman explained: the *money will be given to me (the woman) no matter how much it is*, *or if it is enough or not; that’s my responsibility to manage it…* [and] *make decisions about what foods to buy* (woman, FS1, 2017). However the diversity of foods consumed during meals–that is, the addition or inclusion of fish, other animal-source foods, vegetables or a chili or tomato ‘*sambal*’–was dependent on, or limited by, what women could afford to buy and the foods available to purchase within the village.

Household consumption of fish–and the availability of money to purchase foods–depended on the type and success of men’s fishing activities. Daily judgements were made as to whether harvested fish were sold fresh, dried for sale, or retained for home consumption. While fish consumed at home were most often harvested by the husband, women also purchased low-value small pelagic fish (e.g. anchovy, sardine) or small-medium reef fish (e.g. emperor fish, rabbitfish, grouper), in either fresh or dried form, from village fishers, or received fish as a gift from relatives or friends. Women purchased fish if or when their husband hadn’t caught fish, or was absent from the village on multi-day fishing trips. The availability of fish–and thus income earned–was also affected by the moon phases, with some fishing activities not possible during the full-moon, and the seasons, with fish more abundant but fishing more hazardous during the stormy wet season (grounding fishers for days at a time) and fishing requiring more effort during the calmer dry season.

Other nutrient-dense foods, such as eggs and fruit and vegetables, were available from a small number of vendors in each village. However, without access to refrigeration vendors estimated losing roughly one quarter of their produce to spoilage before it could be sold. The fragility of access to fruit and vegetables was illustrated by the situation in FS2 during our second survey period: the sole vendor had been away from the community for the previous week *so no one has had any vegetables*! (woman, FS2, 2018). Women did have the autonomy to visit the larger and cheaper food markets in Labuan Bajo, however the cost of transport outweighed the small quantities of perishable foods purchased. Further, while it was possible to obtain produce on credit from vendors in the villages, *they would have to pay cash upfront* in Labuan Bajo (woman, FS2, 2018).

The burden and stress of ensuring that a family had sufficient food fell on women. Women *had to cover for [their] husband if he [didn’t] earn enough money and not show to anyone that he doesn’t provide enough* (woman, FS2, 2018). Women used their social capital and networks to secure food: they would obtain food from female relatives or friends, borrow money from their husband’s ‘boss’, or purchase food from kiosks or vendors on credit. These activities aimed at securing staple and alternate foods (e.g. rice, noodles, cooking oil) and, while borrowing money and obtaining foods (and other household goods) on credit were common, this strategy may compound household indebtedness. Other strategies employed by women to manage through times of insufficient food included reducing family–and their own–portions, sending children to eat with relatives, or substituting non-preferred foods for preferred foods (e.g. eggs for fish, noodles for rice). Locally harvested moringa leaves (‘*kelor*’, *Moringa oleifera*) were substituted for other dark leafy greens.

#### Gleaning of marine resources for food and income

Gleaning has been identified as an important source of nutrient-dense marine foods for household consumption in other studies [53, 54]. However, we found that women did not regularly engage in gleaning. One fifth of women respondents reported gleaning in areas immediately adjacent to the village or a short boat trip away, with two-thirds of these women reporting that the items gleaned were solely for household consumption. The most commonly harvested species were sea urchin, long-spined sea urchin, shells, and giant clams. One woman for example gleaned long-spined sea urchin, collecting *enough to make up five plates*, *four to sell in the community and one for [her] family* (woman, FS1, 2018). However, only nine percent of the women surveyed had consumed gleaned species (long-spined sea urchin, sea urchin, giant clam and sea anemone), and then only in the wet season. None of the children had consumed gleaned species in either season.

Women indicated that they generally would only glean if their husband’s fishing was unsuccessful or they weren’t able to obtain fish by other means. Women related the cessation of gleaning to the arrival of their children and thus being in the later stages of pregnancy or caring for young children seemed to lessen the frequency of gleaning. Conversely, younger women spoke of not knowing how to collect and prepare the common long-spined sea urchin, or being afraid of being stung by a stingray.

## Discussion

This paper has presented findings from an exploration of how fish and small-scale fisheries activities contribute to food and nutrition security, focusing on the consumption pathway, in three marine-dependent communities in Komodo District, eastern Indonesia. The study found that the dietary diversity of mothers and their children was below the minimum recommended for a diet with micronutrient adequacy, with less than one quarter of women having adequate dietary diversity in either season. Dietary diversity is an important dimension of dietary quality, with a diverse diet inclusive of nutrient-dense animal-source foods and fruits and vegetables more likely to provide the micronutrients essential for the health, well-being and work capacity of women, and also for the growth, development and long-term health of their children [55, 56]. Other research has shown that women are likely to suffer micronutrient deficiencies when their dietary choice is constrained and centred on a monotonous diet of one or a few staple foods [55]. Campbell, Thorne-Lyman et al. [57] found that Indonesian women of reproductive age consuming a rice-based diet with the addition of only small amounts of fruits and vegetables and animal-based foods were at higher risk of clinical vitamin A deficiency, while national data indicate that 42% of women of reproductive age suffer from anaemia [17]. This suggests that the diets of women in our study may be inadequate to meet their micronutrient needs.

Children’s dietary diversity was similarly low, with only 24% and 42% of children having adequate dietary diversity in the wet and dry seasons respectively. The dietary diversity of infants (6–11 months) and young children (12–17 months) was generally lower than older children, reflecting the progressive introduction of complementary foods until children were served family meals and also beliefs and practices about suitable complementary foods. The WHO recommends exclusive breastfeeding of infants until six months of age, and then continued breastfeeding complemented by the introduction of low-cost locally-available foods which are sufficiently energy- and nutrient-dense, and diverse, until a child reaches two years of age [58]. Poor complementary feeding has been identified as a risk factor for stunting, which, while beginning *in utero*, is most often expressed during early childhood (6–23 months) [19]. We found that 50% of children had stunted growth. Typical complementary foods in low-income countries comprise cereal-based porridge, which are lacking in the micro- and macronutrients necessary to support a child’s growth [59] and thus the addition of nutrient-dense locally-available complementary foods are greatly beneficial. In our study, complementary foods were introduced to most children from five or six months of age and included homemade rice porridge or commercially-prepared infant cereals. However these were not routinely supplemented with animal-source foods such as fish, or vegetables. Further, only half of the children in our study achieved the minimum recommended meal frequency for their age and relevant to their consumption of breastmilk.

Fish is increasingly recognised as offering great potential for inclusion in nutrition-sensitive food-based strategies to increase the quality of children’s diets [2, 3]. Fish are rich in nutrients, including iron and zinc, which are lacking in breastmilk, and ground dried small fish could provide a nutritional boost to the rice-based porridges which are introduced as complementary foods and are consumed in small quantities, given children’s limited stomach capacity [3, 60]. We found that while fish was the most commonly consumed animal-source food by mothers, fish was not introduced to the diets of infants and young children until they approach 18 months or two years of age. This finding differs from that of Bandoh and Kenu [61], who found a higher level of fish consumption among children in fishing communities in Ghana, but similar to Thorne-Lyman, Valpiani et al. [62]’s study of fish-farming households in rural Bangladesh where animal-source foods (fish and meat) were withheld from the diets of infants and young children. Socio-cultural practices, including gender norms, and food taboos are recognised as affecting whom within a household receives different foods, and portions thereof, and, as we found here, this can particularly affect pregnant and lactating women and young children [63]. In our study, as in Thorne-Lyman, Valpiani et al. [62], cultural beliefs about the benefits and harms of fish for children were at play.

Several research programs in south and south-east Asia (e.g. Bangladesh [60]) and sub-Saharan Africa (e.g. Kenya [64], Malawi [65]) have investigated the use of locally-sourced dried small fish powders as part of home-based approaches to dietary diversification for children, with the advantage that there was already recognition of the nutritional value, acceptability and popularity of fish within local cuisine. These studies demonstrate that consumption of fish-enriched porridge has the potential to contribute substantially to children’s daily nutrient requirements. Dewey however cautions that “dietary diversification and enrichment of complementary foods (e.g. with fish powder), while important for the overall nutritional quality of the child’s diet, may be insufficient to completely close the gap for key nutrients such as iron” [66]. The nutrient composition and quality of fish also varies depending on life cycle, food availability, season, and changes in the wider environment, and the processing, storage and cooking method used [11, 67]. Access to technologies to improve the quality of both fresh and dried fish–and to allow mothers to more easily process bony fish–are therefore important. In the study communities, access to ice was limited and fish were observed to be salted and/or dried by processes that reduced the quality of fish. For example, small pelagic fish were laid out to dry on nets spread on the ground and not protected from insects or small livestock. Thus any program seeking to enhance dietary diversity, and dietary quality, through increased consumption of fish–or other nutrient-dense foods–must be culturally-sensitive and contextually-appropriate given the broader food environment.

This food environment encompasses the availability, affordability, convenience and desirability of foods [68]. In our study the availability of nutrient-dense foods was limited by the isolation of the communities from markets and the lack of cold storage which resulted in high rates of spoilage. Nutrient-dense foods were more expensive than the widely-available single-serve packaged and processed foods such as powdered fruit drink sachets and sweet and savoury snack foods. These foods, as well as homemade snack foods, were consumed frequently by mothers and children, suggesting that they were convenient options for busy caregivers and that they were desired by children. While some of these foods were fortified, they were often high in added sugars and/or sodium. In addition, frequently consumed powdered fruit drinks were made with water from unimproved sources. This dietary pattern highlights other important dimensions of diet quality, being moderation of consumption of low-nutrition density foods and foods associated with increased risk for chronic diseases such as diabetes [45]. The literature indicates that this consumption pattern is however typical of low- and middle-income countries progressing through nutrition transition [69], where rapid demographic, social and economic change is accompanied by changes in food systems and lifestyle and dietary patterns such that an increasing proportion of energy is derived from fats and refined carbohydrates [70, 71].

The literature reports an association between various dimensions of women’s empowerment and women’s and children’s nutrition outcomes, although these differ by culture and context and in some cases the causal pathways are still to be elucidated [15, 72, 73]. A woman’s ability to make informed decisions about her own health and the health of her children, including accessing pre- and post-natal care, are essential for her own well-being and that of her children, while women’s control over financial and productive resources can change the composition of household purchases, including towards the consumption of more nutritious foods [74]. Women’s time burden, encompassing traditional household activities and childcare to informal and unpaid activities undertaken in support of the main livelihood activity and formal work, can impact the quantity and quality of care for themselves and their children, including preparation of complementary foods [15, 63, 73]. In our study, mother’s dietary diversity was a strong predictor of children’s dietary diversity, while associations with other dimensions of women’s empowerment including maternal education and a woman having her own income-earning activity were not statistically significant. Women in the study communities were responsible for food provisioning and preparation of family foods, and also managed household monies, however, they had low levels of education and tended to rely on the advice of their mothers and traditional healers over local health workers when it came to child rearing and feeding practices. Ultimately, these responsibilities–and women’s food choices–were mediated by the variability of household income and the broader food environment, in which nutrient-dense foods were less accessible and more expensive.

## Conclusion

This study explored how consumption of fish contributes to the diets and nutrition of women and children in three marine-dependent communities in Komodo District, eastern Indonesia. We found that the diets of women and children were characterised by lower dietary diversity than is recommended for diets with micronutrient adequacy, leaving them vulnerable to hidden hunger. Fish were the main animal-source food consumed by women, yet the introduction of fish to the diets of many infants and young children was delayed. We found that women had pivotal roles in food provisioning at the household level, but dietary quality was affected by a range of other factors including variability in incomes from small-scale fisheries activities, and a food environment in which access to nutrient-dense foods was limited whereas low-nutrition density foods were readily available and convenient. The study provides a snapshot of food consumption at the time of survey (2017–2018) and, while the dietary assessment tool used was not quantitated, the collection of data in two seasons allowed for consideration of seasonal changes in food access, availability and utilisation.

With food security assessments typically focusing on food consumption at the household level, we have drawn attention to important differences in intra-household consumption of nutrient-dense foods by vulnerable household members and identified the delayed introduction of fish to the diets of infants and young children. The research also highlights the importance of widening the net beyond the availability of fish, and addressing each of the pillars of food security in efforts to improve the well-being of fishing communities. In doing so, the study provides important insights for forthcoming nutrition-sensitive interventions in similar coastal communities in Indonesia, and across the Coral Triangle Region.

## Supporting information

S1 Data (PDF)

S2 Data (PDF)

## References

[1] Bene C, Barange M, Subasinghe R, Pinstrup-Andersen P, Merino G, Hemre GI, et al Feeding 9 billion by 2050—Putting fish back on the menu. Food Security. 2015;7(2):261–74. 10.1007/s12571-015-0427-z

[2] Bogard JR, Farook S, Marks GC, Waid J, Belton B, Ali M, et al Higher fish but lower micronutrient intakes: Temporal changes in fish consumption from capture fisheries and aquaculture in Bangladesh. PLoS One. 2017;12(4). Epub April 6, 2017. 10.1371/journal.pone.0175098 28384232 PMC5383130

[3] Thilsted SH, Thorne-Lyman A, Webb P, Bogard JR, Subasinghe R, Phillips M, et al Sustaining healthy diets: The role of capture fisheries and aquaculture for improving nutrition in the post-2015 era. Food Policy. 2016;61:126–31.

[4] Kawarazuka N, Bene C. Linking small-scale fisheries and aquaculture to household nutrition security: an overview. Food Security. 2010;2:343–57.

[5] Fabinyi M, Dressler WH, Pido MD. Fish, trade and food security: moving beyond 'availability' discourse in marine conservation. Human Ecology. 2016:12 Epub December 29, 2016 10.1007/s10745-016-9874-1

[6] Mills DJ, Westlund L, de Graaf G, Kura Y, Willman R, Kelleher K. Under-reported and Undervalued: Small-scale Fisheries in the Developing World In: Pomeroy RS, Andrew NL, editors. Small-scale Fisheries Management: CAB International; 2011 p. 1–15.

[7] FAO, IFAD, UNICEF, WFP, WHO. The State of Food Security and Nutrition in the World 2018: Building climate resilience for food security and nutrition. Rome: Italy: 2018.

[8] Charlton KE, Russell J, Gorman E, Hanich Q, Delisle A, Campbell B, et al Fish, food security and health in Pacific Island countries and territories: a systematic literature review. BMC Public Health. 2016;16:26 10.1186/s12889-015-2663-8 27009072 PMC4806432

[9] Cisneros-Montemayor AM, Pauly D, Weatherdon LV, Ota Y. A global estimate of seafood consumption by coastal indigenous peoples. PLoS One. 2016;11(12):16. 10.1371/journal.pone.0166681 PMC5137875 27918581

[10] Roos N, Wahab A, Chamnan C, Thilsted ST. The role of fish in food-based strategies to combat Vitamin A and mineral deficiencies in developing countries. Journal of Nutrition. 2007;137:1106–9. 10.1093/jn/137.4.1106 17374688

[11] Bogard JR, Thilsted SH, Marks GC, Wahab A, Hossain MAR, Jakobsen J, et al Nutrient composition of important fish species in Bangladesh and potential contribution to recommended nutrient intakes. Journal of Food Composition and Analysis. 2015;42:120–33.

[12] HLPE. Sustainable fisheries and aquaculture for food security and nutrition: A report by the High Level Panel of Experts on Food Security and Nutrition of the Committee on World Food Security. Rome: Italy: 2014.

[13] Miller DD, Welch RM. Food system strategies for preventing micronutrient malnutrition. Food Policy. 2013;42:115–28. 10.1016/j.foodpol.2013.06.008

[14] Michaelsen KF, Hoppe C, Roos N, Kaestel P, Stougaard M, Lauritzen L, et al Choice of foods and ingredients for moderately malnourished children 6 months to 5 years of age. Food and Nutrition Bulletin. 2009;30(3):S343–S404. 19998864 10.1177/15648265090303S303

[15] Smith LC, Ramakrishnan U, Ndiaye A, Haddad L, Martorell R. The importance of women's status for child nutrition in developing countries. IFPRI, 2003 Contract No.: Research Report 131.

[16] Stephenson J, Heslehurst N, Hall J, Schoenaker DA, Hutchinson J, Cade JE, et al Before the beginning: nutrition and lifestyle in the preconception period and its importance for future health. The Lancet. 2018;391:1830–41. Epub April 16, 2018. 10.1016/S0140-6736(18)30311-8 PMC6075697 29673873

[17] Development Initiatives. 2018 Global Nutrition Report: Shining a light to spur action on nutrition. Bristol, UK: Development Initiatives, 2018.

[18] Grantham-McGregor S, Cheung YB, Cueto S, Glewwe P, Richter L, Strupp B, et al Developmental potential in the first 5 years for children in developing countries. Lancet. 2007;369:60–70. 10.1016/S0140-6736(07)60032-4 17208643 PMC2270351

[19] Victora CG, Adair L, Fall C, Hallal PC, Martorell R, Richter L, et al Maternal and child undernutrition: consequences for adult health and human capital. The Lancet. 2008;371:340–57. Epub January 17, 2008. 10.1016/S0140-6736(07)61692-4 18206223 PMC2258311

[20] Thilsted SH. Improved management, increased culture and consumption of small fish species can improve diets of the rural poor In: Burlingame B, Dernini S, editors. Sustainable diets and biodiversity: Directions and solutions for policy, research and action. Rome: Italy: FAO; Biodiversity International; 2012 p. 176–81.

[21] Bene C, Arthur R, Norbury H, Allison EH, Beveridge M, Bush SR, et al Contribution of fisheries and aquaculture to food security and poverty reduction: Assessing the current evidence. World Development. 2016;79:177–96.

[22] Foale S, Adhuri DS, Alino P, Allison EH, Andrew N, Cohen P, et al Food security and the Coral Triangle Initiative. Marine Policy. 2013;38:174–83.

[23] Steenbergen DJ, Clifton J, Visser LE, Stacey N, William Mc. Understanding influences in policy landscapes for sustainable coastal livelihoods. Marine Policy. 2017;82:181–8. 10.1016/j.marpol.2017.04.012

[24] Darling ES. Assessing the effect of marine reserves on household food security in Kenyan coral reef fishing communities. PLoS One. 2014;9(11):21 10.1371/journal.pone.0113614 25422888 PMC4244085

[25] Alva S, Kiersten J, Jacob A, D'Agnes H, Mantovani R, Evans T. Marine protected areas and children's dietary diversity in the Philippines. Population and Environment. 2016;37:341–61. 10.1007/s11111-015-0240-9 26924869 PMC4754318

[26] Burchi F, De Muro P. From food availability to nutritional capabilities: Advancing food security analysis. Food Policy. 2016;60:10–9.

[27] UNICEF. Strategy for Improved Nutrition of Children and Women in Developing Countries. Policy Review. New York: USA: 1990 9 March 1990. Report No.: Contract No.: E/ICEF/1990/L.6.

[28] Quisumbing AR, Brown LR, Feldstein HS, Haddad LJ, Pena C. Women: the key to food security. Washington DC: USA: 1995.

[29] Burke L, Reytar K, Spalding M, Perry A. Reefs at Risk Revisited in the Coral Triangle. Washington USA: 2012.

[30] FAO. State of the World Fisheries and Aquaculture 2018—Meeting the sustainable development goals. Rome, Italy: 2018.

[31] FAO. Fishery and Aquaculture Country Profiles: The Republic of Indonesia 2014 [cited 2016 28 October 2016]. Available from: http://www.fao.org/countryprofiles/index/en/?iso3 = IDN.

[32] Halim A, Wiryawan B, Loneragan NR, Hordyk A, Sondita M, White AT, et al Developing a functional definition of small-scale fisheries in support of marine capture fisheries management in Indonesia. Marine Policy. 2019;100:238–48. Epub December 7, 2018. 10.1016/j.marpol.2018.11.044

[33] FAO. The State of World Fisheries and Aquaculture 2016: Contributing to food security and nutrition for all. Rome: 2016.

[34] Harper S, Zeller D, Hauzer M, Pauly D, Sumaila UR. Women and fisheries: Contribution to food security and local economies. Marine Policy. 2013;39:56–63.

[35] Dey MM, Rab MA, Paraguas FJ, Piumsombun S, Bhatta R, Alam MF, et al Fish consumption and food security: A disaggregated analysis by types of fish and classes of consumers in selected Asian countries. Aquaculture Economics and Management. 2007;9(1–2):98–111.

[36] Needham S, Funge-Smith S. The consumption of fish and fish products in the Asia-Pacific region based on household surveys. Bangkok: Thailand: FAO, 2014 Contract No.: RAP Publication 2015/12.

[37] Kementerian Kelautan dan Perikanan. Produktivitas Perikanan Indonesia: Forum Merdeka Barat 9 Kementerian Komunikasi dan Informatika. 2018.

[38] Estimation of Potential Fish Resources in Fisheries Management Areas, Number 50/KEPMEN-KP/2017 (2017).

[39] National Secretariat of CTI-CFF Indonesia. Indonesia National Plan of Actions of Coral Triangle Initiative on Coral Reefs, Fisheries and Food Security. Jakarta: Indonesia: 2009.

[40] Pemerintah Provinsi Nusa Tenggara Timur, Dewan Ketahanan Pangan, Kementerian Pertanian, World Food Programme. Peta Ketahanan Dan Kerentanan Pangan Nusa Tenggara Timur Tahun 2015. 2015.

[41] SEAMEO RECFON. An Evaluation of the 2012–2015 Maternal & Child Nutrition (MNC) Program. Jakarta: Indonesia: 2016.

[42] WFP, SEAMEO TROPMED. Nutrition Security and Food Security in Seven Districts in NTT Province, Indonesia: Status, Causes and Recommendations for Response. Jakarta: Indonesia: 2010.

[43] Komodo National Park Authority. 25 Year Master Plan for Management Komodo National Park: Book 1 Management Plan. 2000.

[44] Daniel WW, Cross CL. Biostatisics: A Foundation for Analysis in the Health Sciences. Tenth Edition ed USA: John Wiley & Sons; 2013.

[45] FAO, FHI 360. Minimum Dietary Diversity for Women: A Guide to Measurement. Rome: Italy: 2016.

[46] WHO. Indicators for assessing infant and young child feeding practices. Part 2: Measurement. Geneva: Switzerland: 2010.

[47] United Nations. How to weigh and measure children: Assessing the nutritional status of young children in household surveys. New York: USA: 1986.

[48] WHO Expert Consultation. Appropriate body-mass index for Asian populations and its implications for policy and intervention strategies. The Lancet. 2004;363:157–63. 10.1016/S0140-6736(03)15268-3 14726171

[49] WHO. WHO Child Growth Standards: Length/height-for-age, weight-for-age, weight-for-length, weight-for-height and body mass index-for-age: Methods and development. Geneva: Switzerland: 2006.

[50] Pollnac RB, Crawford BR. Assessing Behavioural Aspects of Coastal Resource Use. Rhode Island: USA: University of Rhode Island, 2000 Contract No.: Coastal Resources Centre Coastal Management Report #2226.

[51] Filmer D, Pritchett LH. Estimating wealth effects without expenditute data—or tears: An application to educational enrollments in States of India. Demography. 2001;38(1):115–32. 10.1353/dem.2001.0003 11227840

[52] Cinner JE, McClanahan TR, Wamukota A. Differences in livelihoods, socioeconomic characteristics, and knowledge about the sea between fishers and non-fishers living near and far from marine parks on the Kenyan coast. Marine Policy. 2010;34:22–8. 10.1016/j.marpol.2009.04.003

[53] Kleiber D, Harris LM, Vincent ACJ. Improving fisheries estimates by including women's catch in the Central Philippines. Canadian Journal of Fisheries and Aquatic Sciences. 2014;71(5):656–64. 10.1139/cjfas-2013-0177

[54] Chapman MD. Women's fishing in Oceania. Human Ecology. 1987;15(3):267–88.

[55] Torheim LE, Ferguson EL, Penrose K, Arimond M. Women in resource-poor settings are at risk of inadequate intakes of multiple micronutrients. Journal of Nutrition. 2010;140:2051S–8S. 10.3945/jn.110.123463 20881075

[56] Martin-Prevel Y, Allemand P, Wiesmann D, Arimond M, Ballard T, Deitchler M, et al. Moving Forward on Choosing a Standard Operational Indicator of Women's Dietary Diversity. Rome: Italy: 2015.

[57] Campbell AA, Thorne-Lyman A, Sun K, de Pee S, Kraemer K, Moench-Pfanner R, et al Indonesian women of childbearing age are at greater risk of clinical vitamin A deficiency in families that spend more on rice and less on fruits/vegetables and animal-based foods. Nutrition Research. 2009;29:75–81. 10.1016/j.nutres.2008.12.004 19285596

[58] WHO. Infant and young child feeding: WHO; 2018 [cited 2018 July 3, 2018]. Available from: http://www.who.int/news-room/fact-sheets/detail/infant-and-young-child-feeding.

[59] Dewey KG. The challenge of meeting nutrient needs of infants and young children during the preiod of complementary feeding: An evolutionary perspective. Journal of Nutrition. 2013:5 Epub October 16, 2013. 10.3945/jn.113.182527 24132575 PMC3827643

[60] Bogard JR, Hother AL, Saha M, Bose S, Kabir H, Marks GC, et al Inclusion of small indigenous fish improved nutrition quality during the first 1000 days. Food and Nutrition Bulletin. 2015:1–14. 10.1177/0379572115598885 26297705

[61] Bandoh DA, Kenu E. Dietary diversity and nutritional adequacy of under-fives in a fishing community in the central region of Ghana. BMC Nutrition. 2017;3(2):1–6. Epub January 3, 2017.

[62] Thorne-Lyman A, Valpiani N, Akter R, Baten MA, Genschick S, Karim M, et al Fish and meat are often withheld from the diets of infants 6 to 12 months in fish-farming households in rural Bangladesh. Food and Nutrition Bulletin. 2017;38(3):354–68. 10.1177/0379572117709417 28618837

[63] Pachon H, Simondon KB, Fall ST, Menon P, Ruel MT, Hotz C, et al Constraints on the delivery of animal-source foods to infants and young children: Case studies from five countries. Food and Nutrition Bulletin. 2007;28(2):215–29. 10.1177/156482650702800211 24683681

[64] Konyole SO, Kinyuru JN, Owuor BO, Kenji GM, Onyango CA, Estambale BB, et al Acceptability of amaranth grain-based nutritous complementary foods with Dagaa fish (Rastrineobola argentea) and edible termites (Macrotermes subhylanus) compared to Corn Soy Blend Plus among young children/mother dyads in Western Kenya. Journal of Food Research. 2012;1(3):111–20. 10.5539/jfr.v1n3p111

[65] Hotz C, Gibson RS. Participatory nutrition education and adoption of new feeding practices are associated with improved adequacy of complementary diets among rural Malawian children: a pilot study. European Journal of Clinical Nutrition. 2005;59:226–37. 10.1038/sj.ejcn.1602063 15483634

[66] Dewey KG. Increasing iron intake of children through complementary foods. Food and Nutrition Bulletin. 2007;28(4 (Supplement)):S595–S609. 18297897 10.1177/15648265070284S412

[67] Roos N, Leth T, Jakobsen J, Thilsted SH. High vitamin A content in some small indigenous fish species in Bangladesh: perspectives for food-based strategies to reduce vitamin A deficiency. International Journal of Food Sciences and Nutrition. 2002;53(5):425–37. 10.1080/0963748021000044778 12396468

[68] Herforth A, Ahmed S. The food environment, its effects on dietary consumption and potential for measurement within agriculture-nutrition interventions. Food Security. 2015;7:505–20. Epub May 7, 2015. 10.1007/s12571-015-0455-8

[69] Arimond M, Wiesmann D, Becquey E, Carriquiry A, Daniels MC, Deitchler M, et al Simple food group diversity indicators predict micronutrient adequacy of women's diets in 5 diverse, resource-poor settings. Journal of Nutrition. 2010:11 Epub September 29, 2010. 10.3945/jn.110.123414 20881077 PMC2955880

[70] HLPE. Nutrition and Food Systems. Rome: Italy: 2017.

[71] Ghattas H. Food security and nutrition in the context of the global nutrition transition Technical Paper. Rome: Italy: FAO, 2014.

[72] Malapit HJ, Quisumbing AR. What dimensions of women's empowement in agriculture matter for nutrition in Ghana? Food Policy. 2015;52:54–63. 10.1016/j.foodpol.2015.02.003

[73] Komatsu H, Malapit HJ, Theis S. Does women's time in domestic work and agriculture affect women's and children's dietary diversity? Evidence from Bangladesh, Nepal, Cambodia, Ghana, and Mozambique. Food Policy. 2017;79(256–270). 10.1016/j.foodpol.2018.07.002

[74] Bhagowalia P, Menon P, Quisumbing AR, Soundararajan V. What dimensions of women's empowerment matter most for child nutrition? Washington: USA: Poverty, Health, and Nutrition Division, 2012 Contract No.: 01192.

