# Supplementary material for: Dietary diversity and fish consumption of mothers and their children in fisher households in Komodo District, eastern Indonesia
Source: PLoS One. 2020 Apr 1;15(4):e0230777. doi: 10.1371/journal.pone.0230777 (PMC7112201; doi:10.1371/journal.pone.0230777)
Supplement: S1 Data — (PDF) [file pone.0230777.s001.pdf]

Household Socio-Economic, Food Security and Decision-Making Survey

Questionnaire Code:			
EXP:	*e.g. SB_CL_RT1_06 SB: initials of island CL: initials or first two letters of desa RT No. Code: number allocated to household*		
Period:	January	April / May	Response:<br>YES … 1<br>RESP MOVED … 2<br>RESP COULD NOT BE FOUND … 3<br>RESP DECLINED TO PARTICIPATE FURTHER … 4<br>RESP COMPLETED BUT SPOUSE DECLINED TO PARTICIPATE … 5
Modules:	A, B, C Female head HH	B, C Male head HH	
Completed (see response code):			
Informed Consent:			
Enumerator(s):			
Date:			

INFORMED CONSENT
FEMALE HEAD OF HOUSHOLD
<INSERT REQUIRED TEXT>

Confidential

MODULE A: RESPONDENT AND HOUSEHOLD CHARACTERISTICS ** MODULE TO BE COLLECTED ONCE IN JANUARY

PART 1: RESPONDENT CHARACTERISTICS

EXP	This module asks a series of questions about you, your household, and the livelihood activities that household members participate in.<br>NOTE: The respondent should be the female head of household aged between 18 and 49 years of age and who has lived in the desa for more than 6 months. If multiple women of reproductive age, select the one that has more responsibility for food preparation.		
A1.	How old are you?		Response: age in years
A2a.	What is your marital status?	If 1 - 3, SKIP to A2c	Response:<br>Single (never married) ... 1<br>Partner (but not married) ... 2<br>Engaged ... 3<br>Married ... 4<br>Divorced ... 5<br>Separated ... 6<br>Widowed ... 7
A2b.	How old were you when you first got married?		Response: age in years
A2c.	Are you currently pregnant?		Response:<br>YES ... 1 NO ... 2<br>NOTE: if 1, DON'T NEED RESPONDENT'S HEIGHT AND WEIGHT, ONLY CHILD
A3.	Where were you born?		Response:<br>Prompt for name of desa, island or province
A4a.	What is your ethnicity?		
A4b.	What is your religion?		Response:<br>Islam ... 1<br>Protestant ... 2<br>Catholic ... 3<br>Hindu ... 4<br>Buddhist ... 5<br>Confucian ... 6<br>Other (please specify) ... 7
A5.	How many years have you lived in this desa?		Response: no. of years and/or with months
A6a.	Do you own or rent this house?		Response:<br>Own ... 1<br>Rent ... 2<br>Stay with relatives ... 3<br>Other (please specify) ... 4
A6b.	Do you own a house in another location?		Response:<br>YES ... 1 NO ... 2
	Respondent schooling and literacy		
A7.	What schooling have you completed?		Response:<br>Some primary ... 1<br>Completed primary ... 2<br>Some secondary ... 3<br>Completed secondary ... 4<br>More than secondary ... 5
A8a.	Reading:<br>*Ask respondent to read statement from card*		Response:<br>Can read well ... 1<br>Can read part of sentence ... 2<br>Can't read at all ... 3<br>Blind / visually impaired ... 4
A8b.	Numeracy:<br>*Ask respondent to complete problem from card*		Response:<br>Can solve problem ... 1<br>Takes time to solve problem ... 2<br>Can't solve problem ... 3<br>Blind / visually impaired ... 4

PART 2: HOUSEHOLD CHARACTERISTICS

	Characteristics of house		
EXP	NOTE: *Enumerator to make observations and complete this section*		
B9a.	Construction:		Response:<br>Single storey ... 1<br>Double storey with open lower floor ... 2<br>Double storey with enclosed lower floor ... 3
B9b.	Walls of upper floor:		Response:<br>Natural (e.g. bamboo) ... 1<br>Wooden plank ... 2<br>Tripleks (wooden panels) ... 3<br>Iron, tin or zinc sheets ... 4<br>Brick ... 5<br>Other (please specify) ... 6
B9c.	Walls of lower floor:		Response:<br>Natural (e.g. bamboo) ... 1<br>Wooden plank ... 2<br>Tripleks (wooden panels) ... 3<br>Iron, tin or zinc sheets ... 4<br>Brick ... 5<br>Other (please specify) ... 6
B9d.	Floor of main living spaces:		Response:<br>Earthen ... 1<br>Wooden plank ... 2<br>Cement ... 3<br>Tile ... 4<br>Other (please specify) ... 5

B9e.	Roof:		Response:<br>Natural (e.g. palm leaves) ... 1<br>Iron, zinc or tin sheets ... 2<br>Tile ... 3<br>Other (please specify) ... 4
	Household services		
B10a.	What is your household's main source of lighting or electricity?		Response:<br>Kerosene lamp ... 1<br>Battery powered lamp ... 2<br>HH own solar panel ... 3<br>HH own generator ... 4<br>Purchased from private generator ... 5<br>Village electricity system ... 6<br>Other (please specify) ... 7
B10b.	What is the main source of drinking water for members of your household?<br>*NOTE:*<br>*- "dijual air" refers to a person who sells water from profil tanks supplied with water brought from Labuan Bajo / Menjaga*<br>*- the spring in Pulau Seraya Besar is protected (ineffectively).*<br>*- the wells in all locations are unprotected because open to air*	If 1, SKIP to B8g<br>If 2, SKIP to B8e	Response:<br>Bottled water ... 1<br>Rainwater collection ... 2<br>Dijual air ... 3<br>Protected dug well ... 4<br>Unprotected dug well ... 5<br>Tubewell / bore hole ... 6<br>Spring ... 7<br>Surface water (e.g. stream) ... 8<br>Other (please specify) ... 9
B10c.	How long does it take to go there, get water, and come back?		Response:<br>No of minutes ... number<br>Don't know ... 2
B10d.	Who usually goes to this source to fetch the water for your household?		Response:<br>Adult woman ... 1<br>Adult man ... 2<br>Female child (under 15) ... 3<br>Male child (under 15) ... 4<br>Don't know ... 5
B10e.	If you obtain drinking water from any source other than bottled water, do you treat the water in any way to make it safer to drink?	If NO or DK, SKIP to B8g	Response:<br>YES ... 1<br>NO ... 2<br>Don't know ... 3
B10f.	What do you usually do to the water to make it safer to drink?<br>** select all that apply<br>NOTE:<br>- for boiling, prompt for duration of boiling (rolling boil for at least 5 minutes); if do not boil for 5 minutes enter 7 with "boil"		Response:<br>Boil ... 1<br>Add bleach / chlorine ... 2<br>Strain it through a cloth ... 3<br>Use a water filter (e.g. ceramic / sand) ... 4<br>Solar disinfection ... 5<br>Let it stand and settle ... 6<br>Other (please specify) ... 7<br>Don't know ... 8
B10g.	What is the main source of water used by your household for other purposes, such as cooking and hand washing?		Response:<br>Bottled water ... 1<br>Rainwater collection ... 2<br>Dijual air ... 3<br>Protected dug well ... 4<br>Unprotected dug well ... 5<br>Tube well / bore hole ... 6<br>Spring ... 7<br>Surface water (e.g. stream) ... 8<br>Other (please specify) ... 9
B10h.	On average, how much does your household spend on drinking water each month?		
B10i.	On average, how much does your household spend on other water each month?		
B10j.	What kind of toilet facilities do members of your household usually use?	If 5 or 6, skip to B8l.	Response:<br>Flush/pour flush to:<br>Septic tank ... 1A<br>Pit latrine ... 1B<br>Elsewhere ... 1C<br>Ventilated improved pit latrine ... 2<br>Pit latrine with slab ... 3<br>Pit latrine without slab .. 4<br>Bucket ... 5<br>No facilities / beach ... 6<br>Other (please specify) ... 7
B10k.	Do you share this facility with other households?		Response:<br>YES ... 1 NO ... 2
B10l.	How do you mainly dispose of household rubbish?		Response:<br>Rubbish pit own property ... 1<br>Village rubbish bins / pit ... 2<br>Burn ... 3<br>Just put outside ... 4<br>Put in sea ... 5
	Household assets		
B11.	Does anyone in this household own the following assets?<br>*NOTE: for electronic and mechanical items, must be currently working.*		Response:<br>Fan ... 1<br>Fridge / freezer ... 2<br>Television ... 3<br>Mobile phone ... 4<br>Computer / tablet ... 5<br>Couch / sofa set ... 6<br>Washing machine ... 7<br>Motorbike / scooter ... 8<br>Car ... 9

	Household profile
EXP	Note: *a household is a group of people who usually live together in the same dwelling unit and have common cooking and eating arrangements, and who acknowledge one adult member as the head of the household* (Indonesia DHS 2012).
B12a.	How many people are there in your household?
B12b.	Household members

No.	Name	Gender	Age	Relationship to respondent	Level of schooling	Illness in previous 2 weeks
1	*Transfer respondent's name*			RESPONDENT		
2						
3						
4						
5						
6						
7						
8						
9						
10						

Response:	
Age:	Age in years, and months (for infants and children aged up to five years)
Gender:	Female ... 1 Male ... 2
Relationship to respondent:	Spouse / partner ... 1 Son ... 2 Daughter ... 3 Sibling ... 4 ... Parent ... 5 Grandparent ... 6 Parent-in-law ... 7 Other ... 8
Level of schooling:	Enrolled (i.e. child/youth) ... 1 Some primary ...2 Completed primary ... 3 Some secondary ... 4 Completed secondary ... 5 More than secondary ... 6 Don't know ... 7
Illness in previous 2 weeks:	YES... 1 NO ... 2 DK... 3 EXP. For example fever, cough, diarrhoea

Age if born 1st of	Jan	Feb	Mar	April	May	June	July	Aug	Sep	Oct	Nov	Dec
2017	12 m	11 m	10 m	9 m	8 m	7 m	6 m	N/A	N/A	N/A	N/A	N/A
2016	2 yr / 24 m	1 yr 11 m / 23 m	1 yr 10 m / 22 m	1 yr 9 m / 21 m	1 yr 8 m / 20 m	1 yr 7 m / 19 m	1 yr 6 m / 18 m	1 yr 5 m / 17 m	1 yr 4 m / 16 m	1 yr 3 m / 15 m	1 yr 2 m / 14 m	1 yr 1 m / 13 m
2015	3 yr / 36 m	2 yr 11 m / 35 m	2 yr 10 m / 34 m	2 yr 9 m / 33 m	2 yr 8 m / 32 m	2 yr 7 m / 31 m	2 yr 6 m / 30 m	2 yr 5 m / 29 m	2 yr 4 m / 28 m	2 yr 3 m / 27 m	2 yr 2 m / 26 m	2 yr 1 m / 25 m
2014	4 yr / 48 m	3 yr 11 m / 47 m	3 yr 10 m / 46 m	3 yr 9 m / 45 m	3 yr 8 m / 44 m	3 yr 7 m / 43 m	3 yr 6 m / 42 m	3 yr 5 m / 41 m	3 yr 4 m / 40 m	3 yr 3 m / 39 m	3 yr 2 m / 38 m	3 yr 1 m / 37 m
2013	5 yr / 60 m	4 yr 11 m / 59 m	4 yr 10 m / 58 m	4 yr 9 m / 57 m	4 yr 8 m / 56 m	4 yr 7 m / 55 m	4 yr 6 m / 54 m	4 yr 5 m / 53 m	4 yr 4 m / 52 m	4 yr 3 m / 51 m	4 yr 2 m / 50 m	4 yr 1 m / 49 m

Confidential

	Livelihood activities					
EXP	*Note: a livelihood activity is any activity that a member of the household engages in in exchange for cash, wages, or other benefits (such as for food). Break livelihood activities down as much as possible to include different types of fishing and processing activities. Prompt for gleaning.*					
B13a.	No.	Livelihood activity	Household member(s)	Month(s) of year	Income per month (Rp)	Importance
	1					
	2					
	3					
	4					
	5					
	6					
	7					
	8					
	9					
	10					
	11					
	12					
	Response:					
	Household member(s):	No. from B12b.				
	Month(s) of year:	number month (i.e. 1 – 12)				
	Income per month of activity:	Average income from the activity in the months in which the activity occurs				
	Importance:	Ranking of livelihood activity's importance, with 1 being the most important activity for the household's income				

Confidential

	**Gleaning**		
EXP	**If gleaning not identified as a livelihood activity in B13a, ask B14a. If gleaning identified as a livelihood activity in B14a, SKIP to B14c.**		
B14a.	Does anyone in your household collect marine animals or organisms (e.g. kerang, teripang) from the reef, i.e. berkarang, bamete, nubba?	If NO, SKIP to B15a.	Response:<br>YES … 1 NO … 2
B14b.	Who?		Response:<br>Household member no. from A12b.
B14c.	What portion of the gleaning harvest is for your household's consumption versus sale?		Response:<br>Only for HH consumption … 1<br>Only for sale … 2<br>Usually only consume what can't be sold … 3<br>Other (please specify) … 4
B14d.	What are the 10 most common animals / organisms collected?		
	**Livelihood assets**		
B15a.	Does anyone in your household own the following assets that are used for livelihood activities?<br>NOTE: outright ownership versus under a loan arrangement		Response:<br>Canoe … 1 / Panah … 6<br>Ketinting … 2 / Cool box … 7<br>Motor perahu … 3 / Drying racks … 8<br>Bagan perahu … 4 / Steamer / Fryer … 9<br>Pukat … 5 / Sewing machine … 10
B15b.	Does any member of your household own any animals or livestock, such as chickens, ducks, goats?	If 2, SKIP to BA15d.	Response:<br>YES … 1 NO … 2
B15c.	What type of livestock? ** select all that apply		Response:<br>Chickens … 1 / Goats … 4<br>Rosters … 2 / Cows … 5<br>Ducks … 3 / Fish (aquaculture) … 6
B15d.	Does any member of the household currently grow fruits or vegetables?	If 2, SKIP to B16a	Response:<br>YES … 1 NO … 2
B15e.	What types of fruits and/or vegetables are grown?		
	**Household spending**		
B16a.	On average, how much money does your household spend on food each month?		
B16b.	On average, how much money do you need to pay for other necessities (such as school fees, water, electricity, transport) each month?		
	**Savings and loans**		
B17a.	Does anyone in the household have a bank account?	If 2 or 3, SKIP to B18a.	Response:<br>YES … 1 NO … 2 DK … 3
B17b.	Who has a bank account? ** list multiple as apply		Response:<br>Use family member code from A12b.
B18a.	Does anyone in the household have savings?<br><br>** *Savings refer to cash money or other assets such as gold which can be accessed in times of need.*	If 2 or 3, SKIP to B19a.	Response:<br>YES … 1 NO … 2 DK … 3
B18b.	Who has savings?		Response:<br>Use family member code from A12b.
BA18c.	What form do the savings take?		Response:<br>Cash … 1 / Fishers group … 4<br>Bank account … 2 / Gold / jewellery … 5<br>Savings group … 3 / Other (please specify) … 6
B19.a	Does anyone from your household participate in an arisan?	If 2 or 3 SKIP to B20a.	Response:<br>YES … 1 NO … 2 DK … 3
BA19b.	Who participates in an arisan?		Response:<br>Use family member code from B12a.

Confidential

B19c.	What was the money used for, or what do you plan to use the money for?		
B20a.	Does any member of your household have loans?		Response:<br>YES … 1 NO … 2 DK … 3
B20b.	Details of loans		

No.	Household member	What is the reason for the loan?	Duration	What is the approximate amount of the loan?	Who is the provider of loan?	Do you feel confident that the loan will be repaid in accordance with the borrowing terms?
1.						
2.						
3.						
4.						
5.						

Response:		
	Household member:	Use household member no. from B12b
	Reason for loan:	General description of purpose
	Duration:	Enumerator to classify as SHORT … 1 LONG … 2 depending on loan period of <= 1 year
	Provider of loan:	RELATIVE … 1 BANK … 2 SAVINGS GROUP … 3 FISHERS GROUP … 4 BOSS/COLLECTOR … 5 OTHER (Please specify) … 6 DK … 7
	Confident of repayment:	YES … 1 NO … 2 DK / UNSURE … 3

	Government assistance		
B21a.	Does your household receive any government assistance?		Response:<br>YES … 1 NO … 2 DK / UNSURE … 3
BA21b.	What type of assistance does your household receive, i.e. program name?<br>e.g. RASTRA		

PART 3: INFANT/CHILD HEALTH AND WELL-BEING

EXP.	The next set of questions is about the health and well-being of your youngest child aged between 6 months and 5 years of age.		
C1a.	What is the age in months of [NAME]?		Confirm NAME and AGE from household roster in A12b.
C2a.	Has [NAME] been unwell at all in the last two weeks?<br>*Transfer from B12b – Household profile*	If 1, SKIP to C3a.	Response:<br>NO… 1 YES … 2
C2b.	Did [NAME} have diarrhoea, a fever or a cough?		Response: (may be more than one response)<br>Diarrhoea … 1 Fever … 2 Cough … 3
C2c.	Did you take [NAME] to a health worker for advice or treatment in relation to this illness?		Response:<br>YES … 1 NO … 2
C3a.	Did [NAME] receive breastmilk or infant formula (e.g. Dancow) yesterday?	If 1, SKIP to C3d.	Response:<br>Breastmilk … 1 Infant formula … 2 Neither … 3
C3b.	How old was [NAME] when [NAME} stopped receiving breastmilk?		Response:<br>Age in months
C3c.	Why did you stop feeding [NAME] breastmilk?		
C3d.	How old was [NAME] when you started to feed [NAME] semi-solid or solid foods?		Response:<br>Age in months
C3e.	What type of semi-solid or solid foods did you first introduce to [NAME]?		
C4a.	Does [NAME} currently receive any nutrition supplements?<br>E.g. micronutrient powder	If 1 or 2 SKIP to C5a.	Response:<br>NO … 1 DK … 2 YES … 3
C4b.	If yes, what does [NAME] receive?		

Confidential

MODULE B – HOUSEHOLD DIETARY DIVERSITY AND FOOD SECURITY ** TO BE COLLECTED TWICE – MUSIM BARAT AND MUSIM KERING

EXP.	This module is about you and your household's consumption of food and drinks, and experiences of food security. This module will be repeated again so that we can learn about your household's food consumption and experiences in the wet and dry season. The respondent should be the female head of household aged between 18 and 49 years of age.

PART 1: FOOD CONSUMPTION

EXP	Now I'd like to ask you about the foods and drinks (meals and snacks) that were consumed by all members of your household, whether at home or anywhere else, over the last seven days, that is from [DAY to DAY]; and then the foods and drinks that were consumed by you during the last day and night and then by [NAME], your youngest child, aged between 6 months and five years of age, during the last day and night.<br>Think about the foods that were actually eaten, rather than foods you might like to have eaten or that you might usually eat at this time of year.		
	NOTE: if day of survey is MON → survey period is from previous MON to SUN; TUES → TUES to MON; WED → WED to TUES; THURS → THURS to WED; FRI → FRI to THURS; SAT → SAT to FRI; SUN → SUN to SAT		
D.	1A	First, thinking about **all members of your household**, did any members of your household eat any of these food items on any day in the **last seven days**?	Response:<br>YES ... 1 NO ... 2
	1B	On how many days did anyone eat any of these food items?	No. of days (1-7)
	2	Now, thinking about **only yourself**, did you eat any of these food items **yesterday during the day or night**?	YES ... 1 NO ... 2
	3	Now, thinking about **only [NAME], your youngest child aged between 6 months and five years of age**, did [NAME] eat any of these food items **yesterday during the day or night**?	YES ... 1 NO ... 2

Code: FCS	Code: DD	Food category	Description / examples	1A<br>ALL HOUSEHOLD MEMBERS	1B<br>NUMBER OF DAYS	2<br>RESPONDENT	3<br>CHILD
A	A	Food made from grains	Rice, glutinous rice, noodles, bread or other foods made from grains				
	B	White roots and tubers and plantains	Cassava, white potatoes, taro or any other foods made from white-fleshed roots or tubers, or plantains				
B	C	Pulses (beans, peas and lentils)	Mature beans or peas (fresh or dried seed), lentils or bean/pea products, including tofu and tempeh				
	D	Nuts and seeds	Any tree nut, groundnut/peanut or certain seeds, or nut/seed 'butters' or pastes				
C	E	Milk and milk products	Milk, cheese, yoghurt or other milk products but NOT including butter, ice cream, cream or sour cream				
D	G	Flesh meat	Beef, pork, lamb, goat, rabbit, chicken, duck, other birds or wild game meat				
E	F	Organ meat	Liver, kidney, heart or other organ meats or blood-based foods, including from wild game				
F1.	H	All fish or shellfish	Fresh or dried fish, including canned fish, and other marine animals (e.g. bulubabe, tetehe)				
F2a.		Fish – fresh					
F2b.		If yes, what type of fresh fish was consumed?					
F3a.		Fish – dried					
F3b.		If yes, what type of dried fish was consumed?					
F4a.		Fish - tinned					
F4b.		If yes, what type of tinned fish was consumed?					
F5a.		Shellfish					

Confidential

	F5b.		If yes, what type of shellfish was consumed?					
	F6a.		Other marine animal					
	F6b.		If yes, what type of other marine animal was consumed?					
	G.	I	**Eggs**	Eggs from poultry or any other bird				
	H.	K	**Orange vegetables Vitamin A-rich vegetables, roots and tubers**	Pumpkin, carrots, squash or sweet potatoes that are yellow or orange inside, local examples ...				
	J	J	**Dark green leafy vegetables**	Cassava leaves, spinach, kang kung, kelor, and/or other dark green leaves				
	K	L	**Vitamin A-rich fruits**	Ripe mango, ripe papaya (not oranges)				
	M	M	Other vegetables	Cabbage, corn, eggplant, onion, seaweed				
		N	Other fruits	Avocado, banana, mandarin, pineapple, rambutan, tamarind, watermelon, coconut flesh				
	N	Q	Other oils and fats	Oils, fats or butter added to food or used for cooking, including extracted oils from nuts, fruits and seeds, and all animal fat, coconut milk				
	O	S	Sweet foods	Sugar, honey, jam, cakes, candy, cookies, pastries, cakes and other sweet items (e.g. sugary drinks)				
		R	Savoury and fried snacks	Crisps and chips, fried dough or other fried snacks				
	P	U	**Condiments and spices**	Ingredients used in small quantities for flavour, such as chillies, spices, herbs, fish powder, tomato paste, flavour cubes or seeds				
		V	Other beverages and foods	Tea or coffee if not sweetened, clear broth, alcohol, pickles, olives or similar, coconut water				

PART 2: INFANT / CHILD DIETARY DIVERSITY

EXP:	This question is about the number of meals consumed by [NAME] yesterday during the day and night.		
D2.	How many meals did [NAME] eat yesterday during the day and night?		Response:<br>Number of meals

Confidential

PART 3: HOUSEHOLD FOOD INSECURITY

EXP.	I'd now like to ask you some questions about you and your household's experience in obtaining food. These questions relate to **everyone in your household** and the period of time in the last four weeks (30 days).					
E1.	In the last four weeks … <read statement below><br>If answer is YES …<br>How often did you <summarise statement> ?<br>NOTE: RARELY (once or twice in four weeks) SOMETIMES (3 to 10 times in four weeks) OFTEN (more than 10 times in four weeks)		A.	Response:<br>YES … 1<br>NO … 2<br>**If NO skip to next**	B.	Frequency
	a.	Did you **worry** that your household would not have enough food?				
	b.	Were you or any household member **not able to eat the kinds of foods your preferred** because of a lack of resources?				
	c.	Did you or any household member have to **eat a limited variety of foods** due to a lack of resources?				
	d.	Did you or any household member have to **eat some foods that you really did not want to eat** because of a lack of resources to obtain other types of food?				
	e.	Did you or any household member have to **eat a smaller meal** than you felt you needed because there was not enough food?				
	f.	Did you or any household member have to **eat fewer meals** in a day because there was not enough food?				
	g.	Was there ever **no food to eat** of any kind in your household because of lack of resources to get food?				
	h.	Did you or any household member **go to sleep at night hungry** because there was no enough food?				
	i.	Did you or any household member **go a whole day or night without eating** anything because there was not enough food?				
	Response:<br>E1.B. RARELY … 1 SOMETIMES … 2 OFTEN … 3					

	Acquisition of food		
E2a.	At this time of year, how often do you travel to Labuan Bajo, Warloka, Sape or Bima to purchase fresh food items such as fruit and vegetables for your household's consumption?		Response:<br>ONCE A MONTH … 1 ONCE A WEEK … 3 MORE THAN FOUR TIMES A WEEK … 5<br>TWICE A MONTH … 2 TWO OR THREE TIMES A WEEK … 4 DO NOT … 6
E2b.	At this time of year, how often do you travel to Labuan Bajo, Warloka, Sape or Bima to purchase other food items, such as sugar, flour or beras for your household's consumption?		Response:<br>ONCE A MONTH … 1 ONCE A WEEK … 3 MORE THAN FOUR TIMES A WEEK … 5<br>TWICE A MONTH … 2 TWO OR THREE TIMES A WEEK … 4 DO NOT … 6
	Sharing of food		
E2a.	At this time of year, how often do you give food items to other households?		Response: OFTEN … 1 SOMETIMES … 2 RARELY … 3 DO NOT … 4 ** *NOTE has same meaning as in E11*
E2b.	At this time of year, how often do you receive food items from other households?		Response: OFTEN … 1 SOMETIMES … 2 RARELY … 3 DO NOT … 4

Confidential

MODULE B – HOUSEHOLD DIETARY DIVERSITY AND FOOD SECURITY ** TO BE COLLECTED TWICE – MUSIM BARAT AND MUSIM KERING

EXP.	This module is about you and your household's consumption of food and drinks, and experiences of food security. This module will be repeated again so that we can learn about your household's food consumption and experiences in the wet and dry season. The respondent should be the female head of household aged between 18 and 49 years of age.

PART 1: FOOD CONSUMPTION

EXP	Now I'd like to ask you about the foods and drinks (meals and snacks) that were consumed by all members of your household, whether at home or anywhere else, over the last seven days, that is from [DAY to DAY]; and then the foods and drinks that were consumed by you during the last day and night and then by [NAME], your youngest child, aged between 6 months and five years of age, during the last day and night.<br>Think about the foods that were actually eaten, rather than foods you might like to have eaten or that you might usually eat at this time of year.<br>NOTE: if day of survey is MON → survey period is from previous MON to SUN; TUES → TUES to MON; WED → WED to TUES; THURS → THURS to WED; FRI → FRI to THURS; SAT → SAT to FRI; SUN → SUN to SAT		
D.	1A	First, thinking about **all members of your household**, did any members of your household eat any of these food items on any day in the **last seven days**?	Response:<br>YES ... 1 NO ... 2
	1B	On how many days did anyone eat any of these food items?	No. of days (1-7)
	2	Now, thinking about **only yourself**, did you eat any of these food items **yesterday during the day or night**?	YES ... 1 NO ... 2
	3	Now, thinking about **only [NAME], your youngest child aged between 6 months and five years of age**, did [NAME] eat any of these food items **yesterday during the day or night**?	YES ... 1 NO ... 2

Code FCS	Code DD	Food category	Description / examples	1A ALL HOUSEHOLD MEMBERS	1B NUMBER OF DAYS	2 RESPONDENT	3 CHILD
A	A	Food made from grains	Rice, glutinous rice, noodles, bread or other foods made from grains				
	B	White roots and tubers and plantains	Cassava, white potatoes, taro or any other foods made from white-fleshed roots or tubers, or plantains				
B	C	Pulses (beans, peas and lentils)	Mature beans or peas (fresh or dried seed), lentils or bean/pea products, including tofu and tempeh				
	D	Nuts and seeds	Any tree nut, groundnut/peanut or certain seeds, or nut/seed 'butters' or pastes				
C	E	Milk and milk products	Milk, cheese, yoghurt or other milk products but NOT including butter, ice cream, cream or sour cream				
D	G	Flesh meat	Beef, pork, lamb, goat, rabbit, chicken, duck, other birds or wild game meat				
E	F	Organ meat	Liver, kidney, heart or other organ meats or blood-based foods, including from wild game				
F1.	H	All fish or shellfish	Fresh or dried fish, including canned fish, and other marine animals (e.g. bulubabe, tetehe)				
F2a.		Fish – fresh					
F2b.		If yes, what type of fresh fish was consumed?					
F3a.		Fish – dried					
F3b.		If yes, what type of dried fish was consumed?					
F4a.		Fish - tinned					
F4b.		If yes, what type of tinned fish was consumed?					
F5a.		Shellfish					

Confidential

	F5b.		If yes, what type of shellfish was consumed?					
	F6a.		Other marine animal					
	F6b.		If yes, what type of other marine animal was consumed?					
	G.	I	Eggs	Eggs from poultry or any other bird				
	H.	K	Orange vegetables Vitamin A-rich vegetables, roots and tubers	Pumpkin, carrots, squash or sweet potatoes that are yellow or orange inside, local examples …				
	J	J	Dark green leafy vegetables	Cassava leaves, spinach, kang kung, kelor, and/or other dark green leaves				
	K	L	Vitamin A-rich fruits	Ripe mango, ripe papaya (not oranges)				
	M	M	Other vegetables	Cabbage, corn, eggplant, onion, seaweed				
		N	Other fruits	Avocado, banana, mandarin, pineapple, rambutan, tamarind, watermelon, coconut flesh				
	N	Q	Other oils and fats	Oils, fats or butter added to food or used for cooking, including extracted oils from nuts, fruits and seeds, and all animal fat, coconut milk				
	O	S	Sweet foods	Sugar, honey, jam, cakes, candy, cookies, pastries, cakes and other sweet items (e.g. sugary drinks)				
		R	Savoury and fried snacks	Crisps and chips, fried dough or other fried snacks				
	P	U	Condiments and spices	Ingredients used in small quantities for flavour, such as chillies, spices, herbs, fish powder, tomato paste, flavour cubes or seeds				
		V	Other beverages and foods	Tea or coffee if not sweetened, clear broth, alcohol, pickles, olives or similar, coconut water				

PART 2: INFANT / CHILD DIETARY DIVERSITY

EXP:	This question is about the number of meals consumed by [NAME] yesterday during the day and night.		
D2.	How many meals did [NAME] eat yesterday during the day and night?		Response:<br>Number of meals

Confidential

PART 3: HOUSEHOLD FOOD INSECURITY

EXP.	I'd now like to ask you some questions about you and your household's experience in obtaining food. These questions relate to **everyone in your household** and the period of time in the last four weeks (30 days).					
E1.	In the last four weeks … <read statement below><br>If answer is YES …<br>How often did you <summarise statement> ?<br>NOTE: RARELY (once or twice in four weeks) SOMETIMES (3 to 10 times in four weeks) OFTEN (more than 10 times in four weeks)		A.	Response:<br>YES … 1<br>NO … 2<br>**If NO skip to next**	B.	Frequency
	a.	Did you **worry** that your household would not have enough food?				
	b.	Were you or any household member **not able to eat the kinds of foods your preferred** because of a lack of resources?				
	c.	Did you or any household member have to **eat a limited variety of foods** due to a lack of resources?				
	d.	Did you or any household member have to **eat some foods that you really did not want to eat** because of a lack of resources to obtain other types of food?				
	e.	Did you or any household member have to **eat a smaller meal** than you felt you needed because there was not enough food?				
	f.	Did you or any household member have to **eat fewer meals** in a day because there was not enough food?				
	g.	Was there ever **no food to eat** of any kind in your household because of lack of resources to get food?				
	h.	Did you or any household member **go to sleep at night hungry** because there was no enough food?				
	i.	Did you or any household member **go a whole day or night without eating** anything because there was not enough food?				
	Response:<br>E1.B. RARELY … 1 SOMETIMES … 2 OFTEN … 3					

	Acquisition of food		
E2a.	At this time of year, how often do you travel to Labuan Bajo, Warloka, Sape or Bima to purchase fresh food items such as fruit and vegetables for your household's consumption?		Response:<br>ONCE A MONTH … 1 ONCE A WEEK … 3 MORE THAN FOUR TIMES A WEEK … 5<br>TWICE A MONTH … 2 TWO OR THREE TIMES A WEEK … 4 DO NOT … 6
E2b.	At this time of year, how often do you travel to Labuan Bajo, Warloka, Sape or Bima to purchase other food items, such as sugar, flour or beras for your household's consumption?		Response:<br>ONCE A MONTH … 1 ONCE A WEEK … 3 MORE THAN FOUR TIMES A WEEK … 5<br>TWICE A MONTH … 2 TWO OR THREE TIMES A WEEK … 4 DO NOT … 6
	Sharing of food		
E2a.	At this time of year, how often do you give food items to other households?		Response: OFTEN … 1 SOMETIMES … 2 RARELY … 3 DO NOT … 4 ** *NOTE has same meaning as in E11*
E2b.	At this time of year, how often do you receive food items from other households?		Response: OFTEN … 1 SOMETIMES … 2 RARELY … 3 DO NOT … 4

Confidential

MODULE C: DECISION MAKING, ASSET OWNERSHIP, MEMBERSHIP, ATTITUDES AND KNOWLEDGE

EXP.	The next series of questions are management of your household and attitudes towards certain things.<br>The module is administered to the female head of household (respondent to other modules) and the corresponding male head of household.

MODULE C: DECISION MAKING, ASSET OWNERSHIP, MEMBERSHIP, ATTITUDES AND KNOWLEDGE

EXP.	This next series of questions asks about the management of your household, participation in community activities and your attitudes and knowledge about particular things.			
F1..	In this set of questions, please indicate your level of involvement in decision-making in the specified area on a scale from 0 to 10.<br>If you have no involvement in decisions about the specified area, you would choose 0, if you alone make decisions you would choose 10, but if you share decision-making equally with your spouse or another person (like a parent or parent-in-law) you would choose 5.			
	Are/were you involved in determining:			Response:<br>0 – 10<br><br>0 – no involvement in decision<br>… occasional involvement<br>5 – shared involvement<br>… involvement in most decisions<br>10 – makes decision by self always
	a.	Fishing activities, including things like when, where, and how to fish		
	b.	Other productive activities, such as starting and running a kiosk or small businesses		
	c.	The raising of livestock (e.g. chickens, ducks, goats) for food		
	d.	The type and amount of food in your family		
	e.	Having a source of your own income		
	f.	Management of family money (income from both the husband and wife)		
	g.	Your own health including attending the pustu		
	h.	Decision to continue your own education		
	i.	Decision to use contraception for family planning		
	j.	Female spouse visiting the market in Labuan Bajo		
F2.	I'd now like to ask you about your opinion about managing your household and daily activities.			
	Can you please tell me whether you agree, somewhat agree or disagree with the following statements:			
	a.	My actions are partly because I will get in trouble with someone if I act differently		Response:<br>Completely agree … 1<br>Somewhat agree … 2<br>Disagree completely … 3<br>DK … 4
	b.	I do what I do so others don't think poorly of me		
	c.	I do what I do because I personally think it is the right thing to do		
F3.	In this set of questions, please indicate whether you agree or disagree with the following statements.<br>We are interested in your opinion only, and you don't need to tell us whether you have personally experienced the situation described.			

		A	B	C	D	E	
F3.	a.	In your opinion, a husband is justified in hitting or beating his wife in the following situations:					
		A	B	C	D	E	Response:<br>Agree … 1<br>Disagree … 2<br>DK … 3
		Goes out without telling him	Neglects the children	Argues with him	Refuses sexual intercourse	Burns the food	
	b.	In your opinion, a husband is justified in verbally chastising his wife (ie. shouting at her or calling her derogatory names) in the following situations:					
		A	B	C	D	E	Response:<br>Agree … 1<br>Disagree … 2<br>DK … 3
		Goes out without telling him	Neglects the children	Argues with him	Refuses sexual intercourse	Burns the food	

F4.	This next set of questions is about involvement in community activities and management of your time.			
	a.	Are you the member of any groups in your community?<br>*For example: fisheries producer or market groups, water, microfinance, mutual help or insurance group, trade or business association, civic/charitable group, local government, religious group, other group*		Response:<br>Yes … 1 No … 2<br>If yes, please specify name / type of group
	b.	Do you feel comfortable speaking up in public to help decide on infrastructure (like water supplies, or sea walls) to be built in your community?		Response:<br>No, not at all comfortable … 1<br>Yes, but with some difficulty …. 2<br>Yes, comfortable … 3<br>DK … 4
	c.	*The next question asks you about how satisfied you are with the time you have to yourself to do things you enjoy. Please give your opinion on a scale of 1 to 10, where 1 means that you are not satisfied and 10 means that you are very satisfied. If you are neither satisfied or dissatisfied, this would be in the middle, or 5 on the scale.*<br>How satisfied are you with your time available for leisure activities such as visiting neighbours, watching TV, or practising sports?		Response:<br>1 – 10<br><br>0 – not satisfied<br>10 – completely satisfied

F5.	This next set of questions is about your family's health and well-being.			
	a.	What are some of the things that you can do to stay healthy?<br>*Prompt for additional responses (anything else?)*		Open response:<br>Eat a variety of foods (five food groups)<br>Eat plenty of fruit and vegetables<br>Eat protein (fish, milk, eggs)<br>Limit consumption of sweet, salty and fatty foods<br>Eat breakfast<br>Drink plenty of clean water<br>Wash hands with soap and clean running water<br>Perform adequate physical activity<br>Maintain a healthy weight<br>Not smoke<br>Other (specify)
	b.	What are some of the ways that pregnant and breastfeeding women can stay healthy?<br>*Prompt for additional responses (anything else?)*		Open response:<br>Eat variety of foods<br>Eat protein, iron and calcium rich foods<br>Take folic acid (or foods containing)<br>Limit food high in salt<br>Drink plenty of clean water<br>Limit caffeine consumption<br>Other (specify)
	c.	What are the key moments when you need to wash your hands?<br>*Prompt for additional responses (anything else)?*		Open response:<br>After going to the toilet<br>After cleaning baby's bottom / changing baby's nappy<br>Before preparing / handling food<br>Before feeding a child / eating<br>After handling raw food<br>After handling trash<br>Other (specify)
	d.	Using soap is not important when you wash your hands.		Response:<br>TRUE … 1 FALSE … 2

Confidential

INFORMED CONSENT
MALE HOUSEHOLD HEAD
<INSERT REQUIRED TEXT>

MODULE C: DECISION MAKING, ASSET OWNERSHIP, MEMBERSHIP, ATTITUDES AND KNOWLEDGE

EXP.	This next series of questions asks about management of your household and attitudes towards certain things.			
F1..	In this set of questions, please indicate your level of involvement in decision-making in the specified area on a scale from 0 to 10.<br>If you have no involvement in decisions about the specified area, you would choose 0, if you alone make decisions you would choose 10, but if you share decision-making equally with your spouse or another person (like a parent or parent-in-law) you would choose 5.			
	Are/were you involved in determining:			Response:<br>0 – 10<br><br>0 – no involvement in decision<br>… occasional involvement<br>5 – shared involvement<br>… involvement in most decisions<br>10 – makes decision by self always
	a.	Fishing activities, including things like when, where, and how to fish		
	b.	Other productive activities, such as starting and running a kiosk or small businesses		
	c.	The raising of livestock (e.g. chickens, ducks, goats) for food		
	d.	The type and amount of food in your family		
	e.	Having a source of your own income		
	f.	Management of family money (income from both the husband and wife)		
	g.	Your own health including attending the pustu		
	h.	Decision to continue your own education		
	i.	Decision to use contraception for family planning		
	j.	Female spouse visiting the market in Labuan Bajo		
F2.	I'd now like to ask you about your opinion about managing your household and daily activities.			
	Can you please tell me whether you agree, somewhat agree or disagree with the following statements:			
	a.	My actions are partly because I will get in trouble with someone if I act differently		Response:<br>Completely agree … 1<br>Somewhat agree … 2<br>Disagree completely … 3 DK … 4
	b.	I do what I do so others don't think poorly of me		
	c.	I do what I do because I personally think it is the right thing to do		

F3.	In this set of questions, please indicate whether you agree or disagree with the following statements.<br>We are interested in your opinion only, and you don't need to tell us whether you have personally experienced the situation described.						
	a.	In your opinion, a husband is justified in hitting or beating his wife in the following situations:					
		A	B	C	D	E	Response:<br>Agree … 1<br>Disagree … 2<br>DK … 3
		Goes out without telling him	Neglects the children	Argues with him	Refuses sexual intercourse	Burns the food	
	b.	In your opinion, a husband is justified in verbally chastising his wife (ie. shouting at her or calling her derogatory names) in the following situations:					
		A	B	C	D	E	Response:<br>Agree … 1<br>Disagree … 2<br>DK … 3
		Goes out without telling him	Neglects the children	Argues with him	Refuses sexual intercourse	Burns the food	

F4.	This next set of questions are about involvement in community activities and management of your time.			
	a.	Are you the member of any groups in your community?<br>*For example: fisheries producer or market groups, water, microfinance, mutual help or insurance group, trade or business association, civic/charitable group, local government, religious group, other group*		Response:<br>Yes … 1 No … 2<br>If yes, please specify name / type of group
	b.	Do you feel comfortable speaking up in public to help decide on infrastructure (like water supplies, or sea walls) to be built in your community?		Response:<br>No, not at all comfortable … 1<br>Yes, but with some difficulty …. 2<br>Yes, comfortable … 3<br>DK … 4
	c.	*The next question asks you about how satisfied you are with the time you have to yourself to do things you enjoy. Please give your opinion on a scale of 1 to 10, where 1 means that you are not satisfied and 10 means that you are very satisfied. If you are neither satisfied or dissatisfied, this would be in the middle, or 5 on the scale.*<br>How satisfied are you with your time available for leisure activities such as visiting neighbours, watching TV, or practising sports?		Response:<br>1 – 10<br><br>0 – not satisfied<br>10 – completely satisfied
F5.	This next set of questions is about the foods that your family eats and your family's health.			
	a.	What are some of the things that you can do to stay healthy?<br>*Prompt for additional responses (anything else?)*		Open response:<br>Eat a variety of foods (five food groups)<br>Eat plenty of fruit and vegetables<br>Eat protein (fish, milk, eggs)<br>Limit consumption of sweet, salty and fatty foods

				Eat breakfast<br>Drink plenty of clean water<br>Wash hands with soap and clean running water<br>Perform adequate physical activity<br>Maintain a healthy weight<br>Not smoke<br>Other (specify)
	b.	What are some of the ways that pregnant and breastfeeding women can stay healthy?<br>*Prompt for additional responses (anything else?)*		Open response:<br>Eat variety of foods<br>Eat protein, iron and calcium rich foods<br>Take folic acid (or foods containing)<br>Limit food high in salt<br>Drink plenty of clean water<br>Limit caffeine consumption<br>Other (specify)
	c.	What are the key moments when you need to wash your hands?<br>*Prompt for additional responses (anything else)?*		Open response:<br>After going to the toilet … 1<br>After cleaning baby's bottom / changing baby's nappy … 2<br>Before preparing / handling food … 3<br>Before feeding a child / eating … 4<br>After handling raw food … 5<br>After handling trash … 6<br>Other (specify)
	d.	Using soap is not important when you wash your hands.		Response:<br>TRUE … 1 FALSE … 2

Confidential

A8a. ** convert to Bahasa Indonesia and Bahasa Sama

Fishing is hard work.	
Children work hard at school.	
The child is reading a book.	
Parents love their children.	

A8b.

17 + 6 = ______

5,000 x 8 = _______

Confidential

Measurement of Height and Weight

Questionnaire code: ____________________

Date: ____________________

Please take this card to ________________________to have your height and weight, and the height and weight of your youngest child aged between 6 months of age and 5 years of age recorded. Please complete this by: ________________.

Person	Age (Y/M)	Gender (L/P)	Weight (kg)	Method (TS/N)	Height (cm)	Method (L/S)	Recorded by:
Mother		P					
Child 1							

Thank you.
