# Supplementary material for: Dietary diversity and fish consumption of mothers and their children in fisher households in Komodo District, eastern Indonesia
Source: PLoS One. 2020 Apr 1;15(4):e0230777. doi: 10.1371/journal.pone.0230777 (PMC7112201; doi:10.1371/journal.pone.0230777)
Supplement: S2 Data — (PDF) [file pone.0230777.s002.pdf]

Survei Sosio Ekonomi, Ketahanan Pangan dan Pengetahuan Gizi dan Pengambilan Keputusan Dalam Rumah Tangga

Kode Kuesioner:			
Penjelasan:	*e.g. SB_CL_RT1_06 SB: inisial pulau CL: inisial atau dua huruf awal dari nama desa RT No. Kode: penomoran rumah tangga*		
Periode:	Januari	April/Mei	Tanggapan:<br>Ya ... 1<br>RESP PINDAH ... 2<br>RESP TIDAK DITEMUKAN ... 3<br>RESP MENOLAK BERPARTISIPASI LEBIH LANJUT ... 4<br>RESP DILENGKAPI TAPI PASANGAN MENOLAK BERPARTISIPASI ... 5
Modul:	A, B, C Kepala Keluarga Perempuan	B, C Kepala Keluarga Laki-Laki	
Dilengkapi (lihat kode respon):			
Informed Consent:			
Enumerator:			
Tanggal:			

FORMULIR PENJELASAN DAN PERSETUJUAN

RESPONDENT 1 - WANITA

<INSERT INFORMED CONSENT TEXT>

Confidential

MODUL A: KARATERISTIK RESPONDEN DAN RUMAH TANGGA ** MODUL UNTUK DIKUMPULKAN SEKALI DI JANUARI

Penjelasan	Modul ini berisi rangkaian pertanyaan mengenai Anda, rumah tangga Anda, dan aktivitas mata pencaharian yang dilakukan oleh anggota keluarga Anda. CATATAN : responden haruslah **wanita kepala rumah tangga berusia 18 dan 49 tahun dan telah tinggal di desa selama lebih dari 6 bulan**. Jika terdapat lebih dari satu wanita usia produktif, pilih wanita yang memiliki tanggung jawab lebih dalam menyiapkan makanan.		
A1.	Berapa usia Anda?		Tanggapan: usia dalam tahun
A2a.	Apa status pernikahan Anda?	Jika 1 -3, Loncat ke A2c	Tanggapan:<br>Bujang (belum pernah menikah) ... 1<br>Pasangan (tapi tidak menikah) ... 2<br>Bertunangan ... 3<br>Menikah ... 4<br>Bercerai ... 5<br>Berpisah ... 6<br>Janda ... 7
A2b.	Berapa usia Anda ketika Anda pertama kali menikah?		Tanggapan: umur dalam tahun
A2c.	Apakah Ibu sedang hamil?		Tanggapan:<br>YA ... 1 TIDAK... 2<br>CATATAN: jika 1, TIDAK PERLU TINGGI DAN BERAT BADAN, HANYA ANAK-ANAK
A3.	Dimana Anda lahir?		Tanggapan:<br>Bantu dengan nama desa, pulau atau provinsi.
A4a.	Apa etnis Anda?		
A4b.	Apa agama Anda?		Tanggapan:<br>Islam ... 1<br>Protestan ... 2<br>Katolik ... 3<br>Hindu ... 4<br>Budha ... 5<br>Koghucu ... 6<br>Lainnya (Sebutkan) ... 7
A5.	Berapa lama Anda sudah tinggal di desa?		Tanggapan: : Jumlah tahun dn bulan
A6a.	Apakah Anda memiliki ataumenyewa rumah ini?		Tanggapan:<br>Milik sendiri ... 1<br>Sewa ... 2<br>Dipakai Bersama keluarga ... 3<br>Lainnya (Sebutkan) ... 4
A6b.	Apakah Anda memiliki rumah di tempat lain?		Tanggapan:<br>YA ... 1 TIDAK... 2
	Level Pendidikan dan Literasi Responden		
A7.	Tingkat Pendidikan apa yang sudah Anda selesaikan?		Tanggapan:<br>Tidak tamat SD ... 1<br>Tamat SD ... 2<br>Tidak Tamat SMP/SMA ... 3<br>Tamat SMA ... 4<br>Perguruan Tinggi ... 5
A8a	Membaca:<br>*Minta responden membaca pertanyaan di kartu*		Tanggapan:<br>Bisa baca dengan baik ... 1<br>Bisa baca sebagian kalimat ... 2<br>Tidak bisa baca sama sekali ... 3<br>Buta / Cacat Penglihatan ... 4
A8b	Menghitung:<br>*Minte responden menyelesaikan soal di kartu*		Tanggapan:<br>Bisa pecahkan soal ... 1<br>Butuh waktu pecahkan soal ... 2<br>Tidak dapat pecahkan soal ... 3<br>Buta / Cacat Penglihatan ... 4

BAGIAN 2 : KARATERISTIK RUMAH TANGGA

	Karateristik Rumah		
EXP	CATATAN : Enumerator untuk membuat observasi dan melengkapi sesi ini		
B9a.	Konstruksi:		Tanggapan:<br>1 lantai ... 1<br>Dua lantai dengan lantai bawah terbuka ... 2<br>Dua lantai dengan lantai bawah tertutup ... 3
B9b.	Dinding lantai atas		Tanggapan:<br>Alami (mis. bambu) ... 1<br>Papan kayu ... 2<br>Tripleks (lembar kayu) ... 3<br>Lembar Besi, Alumunium atau Seng ... 4<br>Batako ... 5<br>Lainnya (Sebutkan) ... 6
B9c.	Dinding lantai bawah		Tanggapan:<br>Alami (mis. bambu) ... 1<br>Papan kayu ... 2<br>Tripleks (lembar kayu) ... 3<br>Lembar Besi, Alumunium atau Seng ... 4<br>Batako ... 5<br>Lainnya (Sebutkan) ... 6
B9d.	Lantai ruang utama		Tanggapan:<br>Tanah ... 1<br>Papan kayu ... 2<br>Semen ... 3<br>Keramik ... 4<br>Lainnya (Sebutkan) ... 5

B9e	Atap:		Tanggapan:<br>Alami (cth. daun lontar) ... 1<br>Lembar besi, seng atau timah ... 2<br>Keramik ... 3<br>Lainnya (sebutkan) ... 4
	Pelayanan Rumah Tangga		
B10a	Apa sumber utama penerangan atau listrik rumah tangga Anda?		Tanggapan:<br>Lampu minyak tanah ... 1<br>Lampu bertenaga baterai ... 2<br>Panel surya milik pribadi... 3<br>Generator milik pribadi ... 4<br>Membeli dari generator swasta ... 5<br>Sistem listrik desa ... 6<br>Lainnya (sebutkan) ... 7
B10b	Apa sumber utama air minum dari anggota rumah tangga Anda?<br>*CATATAN :*<br>- *"Air dijual" merujuk pada orang yang menjual air dari profil tank yang menyediakan air dari Labuan Bajo/Menjaga*<br>- *Sumber Mata Air di Pulau Seraya Besar adalah tertutup (secara tidak efektif)*<br>- *Sumur di semua lokasi adalah tidak terlindung sebab terbuka pada udara*	Jika 1, Loncat ke B8g<br>Jika 2, Loncat ke B8e	Tanggapan:<br>Air kemasan ... 1<br>Air dijual ... 2<br>Tampungan air hujan ... 3<br>Sumur tertutup ... 4<br>Sumur terbuka... 5<br>Sumur bor ... 6<br>Mata air ... 7<br>Air permukaan (mis. aliran air) ... 8<br>Lainnya (sebutkan) ... 9
B10c	Berapa lama waktu yang dibutuhkan untuk pergi mengambil air dan kembali?		Tanggapan:<br>Jumalah waktu<br>Tidak tahu ... 2
B10d	Siapa yang biasa pergi mengambil air untuk rumah tangga anda?		Tanggapan:<br>Wanita Dewasa ... 1<br>Pria Dewasa ... 2<br>Anak Perempuan (usia di bawah 15) ... 3<br>Anak Laki-Laki (usia di bawah 15) ... 4<br>Tidak tahu ... 5
B10e	Jika Anda mengambil ar minum dari sumber lain selain air kemasan, apakah Anda memberi perlakuan pada air sebelum meminumnya?	Jika TIDAK atau TIDAK TAHU, loncat ke A8g	Tanggapan:<br>YA ... 1<br>TIDAK... 2<br>TIDAK TAHU ... 3
B10f	Apa yang biasanya Anda lakukan agar air tersebut lebih aman untuk diminum?<br>** pilih semua yang dilakukan<br>CATATAN :<br>- Untuk air yang direbus, tanyakan lama proses perebusan (air mendidh jika direbus hingga setidaknya 5 menit, jika tidak mendidh, tulis 7 dengan "rebus"		Tanggapan:<br>Rebus ... 1<br>Tambahkan kaporit... 2<br>Saring menggunakan kain ... 3<br>Menggunakan saring air (mis. keramik/pasir)... 4<br>Disinfeksi tenaga surya ... 5<br>Biarkan terendap ... 6<br>Lainnya (sebutkan) ... 7<br>Tidak tahu ... 8
B10g	Apa sumber utama air lainnya di rumah Anda untuk tujuan lain, sperti masak dan mencuci tangan?		Tanggapan:<br>Air kemasan ... 1<br>Tampungan air hujan ... 2<br>Air dijual ... 3<br>Sumur tertutup ... 4<br>Sumur terbuka... 5<br>Sumur bor ... 6<br>Mata air ... 7<br>Air permukaan (mis. aliran air) ... 8<br>Lainnya (sebutkan) ... 9
B10h	Rata-rata, berapa banyak yang rumah tangga Anda habiskan untuk air minum tiap bulan?		
B10i	Rata-rata, berapa banyak yang rumah tangga Anda habiskan untuk air lainnya tiap bulannya?		
B10j.	Fasilitas toilet apa yang anggota rumah tangga Anda biasanya gunakan?	Jika 5 atau 6, loncat ke B8l	Tanggapan:<br>Mengalir/disiram ke:<br>Septic tank ... 1A<br>Galian jamban ... 1B<br>Tempat lain ... 1C<br>Ventilated improved pit latrine ... 2<br>Pit latrine with slab ... 3<br>Pit latrine without slab .. 4<br>Ember ... 5<br>Tidak ada fasilitas/pantai... 6<br>Lainnya (sebutkan) ... 7
B10k	Apakah Anda berbagi fasilitas ini dengan rumah tangga lain?		Tanggapan: YA ... 1 TIDAK... 2
B10l	Kemana Anda umumnya membuang sampah dari rumah tangga Anda?		Tanggapan:<br>Lubang sampah milik sendiri ... 1<br>Tong/Lubang sampah desa ... 2<br>Bakar ... 3<br>Letakkan di luar ... 4<br>Buang ke laut ... 5
	Aset – aset Rumah Tangga		
B11	Apakah anggota keluarga Anda memiliki aset-aset berikut ini?<br>CATATAN : Untuk alat elektronik dan mekanis, haruslah yang masih bisa dipakai		Tanggapan:<br>Kipas angin ... 1<br>Kulkas ... 2<br>Televisi ... 3<br>Telepon genggam ... 4<br>Komputer / tablet ... 5<br>Set sofa ... 6<br>Mesin Cuci ... 7<br>Sepeda Motor... 8<br>Mobil ... 9

	Profil Rumah Tangga
EXP	Catatan: sebuah rumah tangga adalah sekelompok orang yang biasanya tinggal bersama dalam satu unit tempat tinggal dan memiliki peraturan masak dan makan Bersama, dan menunjuk satu anggota keluarga yang dewasa sebagai kepala keluarga. (Indonesia DHS 2012).
B12a.	Ada berapa anggota rumah tangga?
B12b.	Anggota Rumah Tangga

No.	Nama	Umur	Jenis Kelamin	Hubungan dengan responden	Tingkat Pendidikan	Sakit dalam dua minggu terakhir
1				RESPONDEN		
2						
3						
4						
5						
6						
7						
8						
9						
10						

Tanggapan:	
Usia:	Usia dalam tahun dan dalam bulan (untuk bayi dan anak-anak usia 5 tahun)
Jenis Kelamin:	Perempuan... 1 Laki-laki... 2
Hubungan dengan responden:	Pasangan ... 1 Anak Laki-Laki ... 2 Anak Perempuan ... 3 Saudara Kandung ... 4 Orangtua ... 5 ... Kakek/Nenek ... 6 Mertua ... 7 Lainnya ... 8
Level Pendidikan:	Bersekolah (anak/pemuda) ... 1 Tidak tamat SD ... 2 Tamat SD ... 3 Tidak tamat SMP/SMA... 4 Tamat SMA ... 5 Perguruan Tinggi ... 6 Tidak Tahu... 7
Sakit dalam dua minggu terakhir:	YA... 1 TIDAK... 2 TT... 3 Penjelasan. Contoh : demam, batuk, diare

Usia jika lahir pada tanggal 1 bulan	Jan	Feb	Mar	April	Mei	Jun	Jul	Agu	Sep	Okt	Nov	Des
2017	12 m	11 m	10 m	9 m	8 m	7 m	6 m	N/A	N/A	N/A	N/A	N/A
2016	2 yr / 24 m	1 yr 11 m / 23 m	1 yr 10 m / 22 m	1 yr 9 m / 21 m	1 yr 8 m / 20 m	1 yr 7 m / 19 m	1 yr 6 m / 18 m	1 yr 5 m / 17 m	1 yr 4 m / 16 m	1 yr 3 m / 15 m	1 yr 2 m / 14 m	1 yr 1 m / 13 m
2015	3 yr / 36 m	2 yr 11 m / 35 m	2 yr 10 m / 34 m	2 yr 9 m / 33 m	2 yr 8 m / 32 m	2 yr 7 m / 31 m	2 yr 6 m / 30 m	2 yr 5 m / 29 m	2 yr 4 m / 28 m	2 yr 3 m / 27 m	2 yr 2 m / 26 m	2 yr 1 m / 25 m
2014	4 yr / 48 m	3 yr 11 m / 47 m	3 yr 10 m / 46 m	3 yr 9 m / 45 m	3 yr 8 m / 44 m	3 yr 7 m / 43 m	3 yr 6 m / 42 m	3 yr 5 m / 41 m	3 yr 4 m / 40 m	3 yr 3 m / 39 m	3 yr 2 m / 38 m	3 yr 1 m / 37 m
2013	5 yr / 60 m	4 yr 11 m / 59 m	4 yr 10 m / 58 m	4 yr 9 m / 57 m	4 yr 8 m / 56 m	4 yr 7 m / 55 m	4 yr 6 m / 54 m	4 yr 5 m / 53 m	4 yr 4 m / 52 m	4 yr 3 m / 51 m	4 yr 2 m / 50 m	4 yr 1 m / 49 m

Confidential

B13a.	**Aktivitas Mata Pencaharian**					
Penjelasan	Aktifitas mata pencaharian adalah aktifitas yang dilakukan anggota rumah tangga dengan uang tunai, upah atau keuntungan lain (seperti makanan) sebagai gantinya.<br>Rincikan aktifitas mata pencaharian sebanyak mungkin untuk melibatkan jenis aktifitas penangkapan ikan dan pengolahan yang berbeda. Tanyakan mengenai aktifitas *meti*					
	No.	Aktifitas Mata Pencaharian	Anggota Keluarga	Bulan dalam tahun	Pendapatan per bulan (Rp)	Kepentingan
	1					
	2					
	3					
	4					
	5					
	6					
	7					
	8					
	9					
	10					
	Tanggapan:	Anggota Keluarga:	Urutan dariBA12a.			
		Bulan dalam tahun:	Tiga huruf pertama dari nama bulan			
		Pendapatan tiap bulan dari aktifitas :	Rata-rata pendapatan per bulan ketika aktifitas tersebut dijalankan			
		Kepentingan :	Urutan pentingnya aktifitas mata pencaharian, 1 jika merupakan aktofotas paling penting			

Confidential

	Meti		
	Jika *meti* tidak diidentifikasi sebagai aktifitas mata pencaharian di B13a, tanyakan B14a, jika *meti* diidentifikasi sebagai aktifitas mata pencaharian di B13a, loncat ke B14c		
B14a.	Apakah anggota rumah tangga Anda ada yang mengumpulkan hewan laut (contoh kerang, teripang) dari karang seperti meting atau nubba?	Jika tidak, loncat ke B15A	Tanggapan:<br>YA ... 1 TIDAK... 2
B14b.	Siapa?		Tanggapan:<br>Urutan anggota keluarga dari A12b
B14c.	Berapa porsi dari hasil tangkapan yang digunakan sebagai konsumsi rumah tangga dibandingkan dengan yang dijual?		Tanggapan:<br>Hanya untuk konsumsi RT ... 1<br>Hanya untuk jual ... 2<br>Hanya mengonsumsi sisa jualan yang tidak laku ... 3<br>Lainnya (Sebutkan) ... 4
B14d.	Apakah 10 hewan paling utama yang diambil?		
	Aset Mata Pencaharian		
B15a.	Apakah ada di rumah tangga Anda yang memiliki beberapa aset berikut yang digunakan untuk aktifitas mata pencaharian?<br>** membeli secara tunai atau dengan cicilan		Tanggapan:<br>Sampan ... 1<br>Ketinting ... 2<br>Motor perahu ... 3<br>Bagan perahu ... 4<br>Pukat ... 5<br>Panah ... 6<br>Mesin ... 7<br>Boks Ikan ... 8<br>Pengering / Penggoreng... 9<br>Mesin jahit ... 10
B15b.	Apakah ada anggota rumah tangga Anda yang memiliki hewan atau ternak, seperti ayam, bebek, atau kambing?	Jika 2, loncat ke B15d	Tanggapan:<br>YA ... 1 TIDAK... 2
A15c.	Jenis ternak apa? ** pilih semua yang berlaku		Tanggapan:<br>Ayam ... 1<br>Ayam Jantan ... 2<br>Bebek ... 3<br>Kambing ... 4<br>Sapi ... 5<br>Ikan (akuakultur) ... 6
A15d.	Apakah ada anggota rumah tangga yang menanam buah atau sayuran?	Jika 2, loncat ke B16a	Tanggapan:<br>YA ... 1 TIDAK... 2
A15e.	Jenis buah atau sayuran apa yang ditanam?		
	Pengeluaran Rumah Tangga		
B16a.	Dalam rata-rata, berapa banyak pengeluaran rumah tangga Anda untuk makan tiap bulannya?		
B16b.	Dalam rata-rata, berapa banyak uang yang Anda butuhkan untuk, membayar kebutuhan lain (seperti uang sekolah, air, listrik, transport) tiap bulan?		
	Tabungan dan Pinjaman		
B17a.	Apakah ada di rumah tangga Anda ada yang memiliki rekening bank?	Jika 2 atau 3, loncat ke B18a	Tanggapan:<br>YA ... 1 TIDAK... 2 TT ... 3
B17b.	Siapa yang memiliki rekening bank? ** daftarkan semua yang memliki		Tanggapan:<br>Gunakan kode anggota keluarga dari A12b.
B18a.	Apakah ada di rumah tangga Anda yang memiliki tabungan?<br>**tabungan merujuk pada uang tunai atau aset-aset seperti emas yang bisa diakses jika dibutuhkan*	Jika 2 atau 3 loncat ke B19a	Tanggapan:<br>YA ... 1 TIDAK... 2 TT ... 3
B18b.	Siapa yang memiliki tabungan?		Tanggapan:<br>Gunakan kode anggota keluarga dari A12b.
B18c.	Dalam bentuk apa tabungan tersebut?		Tanggapan:<br>Uang Tunai ... 1<br>Rekening bank ... 2<br>Kelompok tabungan ... 3<br>Kelompok nelayan ... 4<br>Emas/perhiasan ... 5<br>Lainnya (sebutkan) ... 6
B19.a	Apakah ada di rumah tangga Anda yang berpartisipasi dalam arisan?	Jika 2 atau 3 loncat ke B20a	Tanggapan:<br>YA ... 1 TIDAK... 2 TT ... 3
B19b.	Siapa yang berpartisipasi dalam arisan?		Tanggapan:<br>Gunakan kode anggota rumah tangga dari A12a.

B19c.	Uang tersebut digunakan untuk apa, atau Anda berencana menggunakan uang itu untuk apa?		
B20a.	Apakah ada anggota rumah tangga Anda yang memiliki pinjaman?		Tanggapan:<br>YA ... 1 TIDAK... 2 TT ... 3

B20b. Rincian Pinjaman

No.	Anggota Keluarga	Apa alasan peminjaman tersebut?	Lama	Berapa jumlah pinjaman tersebut?	Siapa yang memberikan pinjaman?	Apakah anda merasa yakin bahwa pinjaman dapat dikembalikan sesuai dengan perjanjian peminajman?
1.						
2.						
3.						
4.						
5.						

Tanggapan:		
	Anggota Keluarga	Gunakan kode anggota rumah tangga dari AB12b
	Alasan meminjam	Deskripsi umum dari tujuan
	Lama	Enumerator untuk mengklasifikasi sebagai SINGKAT ... 1 LAMA .... 2 Tergantung pada waktu peminjaman <= 1 tahun
	Penyedia pinjaman	SAUDARA ... 1 BANK ... 2 KELOMPOK TABUNGAN ... 3 KELOMPOK NELAYAN ... 4 BOSS/PENGUMPUL ... 5 LAINNYA (Sebutkan) .... 6 TT ... 7
	Keyakinan untuk Pengembalian	YA ... 1 TIDAK... 2 TT / TIDAK YAKIN ... 3

	Bantuan Pemerintah		
B21a.	Apakah rumah tangga Anda menerima bantuan pemerintah?		Tanggapan:<br>YA ... 1 TIDAK... 2 TT / TIDAK YAKIN ... 3
BA21b.	Jenis bantuan apa yang rumah tangga Anda terima? (nama program) contoh RASTRA		

BAGIAN 3 : KESEHATAN BAYI/BALITA

EXP.	Rangkaian Pertanyaan beriKut adalah tentang kesehatan dan pangan yang dikonsumsi oleh **anak paling muda yang berusia 6 bulan hingga 5 tahun** di keluarga Anda.		
C1a.	Berapa usia dari [NAMA]?		Confirm NAME and AGE from household roster in A12b.
C2a.	Apakah [NAMA] mengalami sakit dalam dua minggu terakhir?<br>*Transfer dari B12b – Profil RT*	Jika 1 loncat ke C3a	Tanggapan:<br>TIDAK ... 1 YA... 2
C2b.	Apakah [NAMA] mengalami diare, demam atau batuk?		Tanggapan: (bisa lebih dari satu tanggapan<br>Diare ... 1 Demam .... 2 Batuk 3
C2c.	Apakah Anda membawa [NAMA] ke petugas kesehatan untuk mencari pengobatan atas penyakitnya?		Tanggapan:<br>YA ... 1 TIDAK... 2
C3a.	Apakah [NAMA] diberi ASI atau susu formula (Dancow) kemarin?	Jika 1, loncat ke C3d	Tanggapan:<br>ASI ... 1 Susu formula .... 2 Tidak keduanya ... 3
C3b.	Umur berapa [NAMA] berhenti diberi ASI?		Response:<br>Age in months
C3c.	Kenapa Anda berhenti memberi [NAMA] ASI?		
C3d.	Umur berapa [NAMA] ketika Anda memberinya makanan semi-padat atau padat?		Tanggapan:<br>Umur dalam bulan
C3e.	Makanan semi-padat atau padat apa yang pertama Anda beri untuk [NAMA]?		
C4a.	Apakah [NAMA] sekarang ini mengonsumsi suplemen gizi? Seperti bubuk micronutrient?	Jika 1 atau 2, loncat ke C5a	Tanggapan:<br>TIDAK ... 1 TT ..... 2 YA ... 3
C4b.	Jika YA, apa yang dikonsumsi [NAMA]?		

EXP.	Modul ini adalah tentang konsumsi pangan dan minuman dan pengalaman-pengalaman ketahanan pangan Anda dan rumah tangga Anda. Modul ini akan diulangi lagi sehingga kami bisa mengetahui konsumsi dan pengalamann anda pada musim hujan dan musim kering. Responden haruslah kepala rumah tangga perempuan berusia 18 – 49 tahun.

BAGIAN 1: KONSUMSI PANGAN

EXP	Saya ingin Anda mendeskripsikan pangan (makanan dan kudapan) yang dikonsumsi seluruh anggota keluarga Anda, entah di rumah ataupun di mana saja, selama tujuh hari terakhir, yaitu dari [HARI ke HARI]; dan makanan yang Anda konsumsi pada kemarin siang dan malam, dan oleh [NAMA], anak Anda yang paling muda berusia antara 6 bulan hingga 5 tahun, pada kemarin siang dan malam. Pikirkan makanan yang betul-betul Anda makan dan bukan makanan yang pernah Anda makan atau biasa Anda makan pada musim-musim saat ini.<br>CAT : jika hari survey adalah SEN □ periode survey adalah dari SEN ke MING sebelumnya; SEL → SEL ke SEN; RAB → RAB ke SEL; KAM → KAM ke RAB; FRI → JUM ke KAM; SAB → SAB ke JUM; MING → MING ke SAB		
D	1A	Pertama, pikirkan semua anggota keluarga Anda, Apakah anggota keluarga Anda ada yang mengonsumsi makanan ini pada tujuh hari terakhir?	Tanggapan<br>YA .... 1 TIDAK ... 2
	1B	Berapa hari anggota keluarga Anda mengonsumsi makanan-makanan ini?	Jumlah hari (1 – 7)
	2	Sekarang pikirkan diri Anda sendiri, apakah Anda mengonsumsi makanan ini pada kemarin siang atau malam?	YA .... 1 TIDAK ... 2
	3	Sekarang pikirkan hanya [NAMA], anak termuda anda yang berusia 6 bulan hingga 5 tahun, apakah [NAMA] mengonsumsi makanan ini kemarin siang atau malam?	YA .... 1 TIDAK ... 2

					1A	1B	2	3
	KODE		Kategori Pangan	Deskripsi /Penjelasan	SEMUA ANGGOTA KELUARGA	JUMLAH HARI	RESPONDEN	ANAK
	FCS	DD						
	A	A	Bahan Pangan dari gandum	Nasi, beras ketan, mie, roti makanan lain yang terbuat dari gandum				
		B	Umbi putih dan umbi dan pisang tanduk	Ubi kayu, ubi outih,, kentang, atau makanan laun yang terbuat dari akar berdaging putih atau umbi atau pisang tanduk.				
		C	Kacang – kacangan (kacang merah, kacang polong, kacang lentil)	Kacang polong (segar atau kering, lentil atau produk kacang lainnya termasuk tempe/tahu				
		D	Sejenis kacang tanah dan biji-bijian	Kacang tanah, kacang mete, pasta atau olesan kacang-kacangan				
		E	Susu dan produk susu	Susu,keju, yogurt, atau produk susu lain tapi TIDAK termasuk mentega, es krim, krim atau krim asam				
		G	Daging	Daging sapi, daging babi, daging domba, daging kambing , daging kelinci, daging bebek, burung laing, atau daging hewan liar lain.				
		F	Daging Organ	Hati, ginjal, jantung atau organ lainnya, atau makan berbahan dasar darah, termasuk dari hewan liar				
		H	Semua ikan atau hewan laut	Ikan segar atau kering, termasuk ikan kaleng atau hewan laut lain (bulu babi, tatehe)				
			Ikan - Segar					
			Jika ya, jenis ikan segar apa yang Anda makan?					
			Ikan – Kering					
			Jika ya, jenis ikan kering apa yang					

Confidential

			Anda makan?					
			Ikan - Kaleng					
			Jika ya, jenis ikan kaleng apa yang Anda makan?					
			Hewan Laut					
			Jika ya, jenis hewan laut apa yang Anda makan?					
			Hewan laut lainnya					
			Jika ya, jenis hewan laut lainnya apa yang Anda makan?					
			Telur	Telur dari unggas atau burung lainnya				
			Akar, umbi atau sayuran kaya Vitamin A	Labu, wortel, labu kuning, ubi jalar yang berwarna kuning atau oranye di dalam, contoh local …				
			Sayuran berdaun hijau tua	Daun ubi, sawi, daun labu, daun ubi jalur, kangkong, bayam				
			Buah-buahan kaya Vitamin A	Manga masak, papaya masak (bukan oranye)				
			Sayuran lain	Kol, jagung, terung, bawang, rumput laut				
			Buah-buahan lain	Alpukat, pisang, jeruk, nanas, rambutan, asam, semangka, daging kelapa				
			Minyak dan lemak lain	Minyak, lemak, atau mentega yang ditambahkan pada makanan atau digunakan saat memeasak termasuk minyak yang diekstrak dari kacang, buah dan biji-bijian, dan semua lemak hewan, santan.				
			Kudapan manis	Gula, madu, selai, permen, kue, pastry, keik, dan makanan manis lain (minuman manis)				
			Kudapan gurih dan goreng-gorengan	Keripik, gorengan, atau kudapan gorengan lain				
			Bumbu dan penyedap rasa	Bahan yang digunakan dalam jumlah sedikit untuk memberi rasa, sepeti cabai, bumbu-bumbu, bawang putih, daun-daunan, bubuk ikan, saus tomat, penyedap rasa, atau biji-bijian				
			Minuman dan makanan lain	Kopi atau teh tawar, kaldu bening, alcohol, acar, zaitun dan sejenisnya, air kelapa				

BAGIAN 2: KERAGAMAN DIET ANAK-ANAK DAN BAYI

EXP:	Pertanyaan ini tentang makanan yang dikonsumsi [NAMA] kemarin siang dan malam		
D2.	Berapa banyak mkaan yang dikonsumsi [NAMA] kemarin siang dan malam?		Tanggapan:<br>Jumlah makanan

Confidential

BAGIAN 3: KERAWANAN PANGAN RUMAH TANGGA

EXP.	Sekarang Saya ingin bertanya pada Anda beberapa pertanyaan mengenai pengalaman Anda dan keluarga dalam mendapatkan pangan. Pertanyaan ini berkaitan dengan **setiap orang di rumah tangga Anda** dengan periode waktu dalam empat minggu terakhir (30 hari).					
E1.	Dalam empat minggu terakhir … <bacakan pertanyaan berikut><br>Jika jawabannya YA …<br>Seberapa sering Anda <ringkaskan pernyataan> ?<br>CATATAN: JARANG (satu atau dua kali dalam 4 minggu) KADANG (3 sampai 10 kali dalam 4 minggu SERING (lebih dari 10 kali dalam 4 minggu)		A.	Tanggapan:<br>YA … 1<br>TIDAK… 2<br>Jika TIDAK LONCAT ke berikutnya	B.	Frekuensi
	a.	Apakah Anda **khawatir** jika keluarga Anda tidak akan memiliki cukup makanan?				
	b.	Apakah Anda atau anggota keluarga yang lain **tidak mampu untuk mengonsumsi jenis makanan yang diinginkan** karena kurang sumber daya?				
	c.	Apakah Anda atau anggota keluarga yang lain harus **makan variasi makanan yang terbatas** karena kurangnya sumberdaya?				
	d.	Apakah Anda atau anggota keluarga Anda harus **makan makanan yang sebenarnya tidak ingin Anda makan** karena kurangnya sumberdaya untuk mendapatkan jenis makanan lainnya?				
	e.	Apakah Anda atau anggota keluarga Anda harus **makan makanan lebih sedikit** dari yang Anda pikir Anda butuhkan karena kurangnya makanan?				
	f.	Apakah Anda atau anggota keluarga yang lain harus **makan makanan lebih sedikit** dalam satu hari karena kurangnya makanan?				
	g.	Pernahkan **tidak ada makanan apapun untuk dimakan** di keluarga anda karena kurangnya sumberdaya untuk mendapatkan makanan?				
	h.	Apakah Anda atau anggota keluarga yang lain **pergi tidur malam dalam keadaan lapar** karena tidak ada cukup makanan?				
	i.	Apakah Anda atau anggota keluarga yang lain **beraktifitas seharian atau semalaman tanpa makan** karena tidak ada makanan yang cukup?				
	Tanggapan:<br>E1.B. JARANG … 1 KADANG … 2 SERING … 3					

	Perolehan makanan				
E2a.	Pada waktu seperti ini, seberapa sering Anda bepergian ke Labuan Bajo, Warloka, Sape atau Bima untuk membeli makanan segar seperti buah dan sayur untuk konsumsi rumah tangga Anda?		Tanggapan:<br>SEKALI SEBULAN … 1<br>DUA KALI SEBULAN … 2	SEKALI SEMINGGU … 3<br>DUA ATAU TIGA KALI SEMINGGU… 4	LEBIH DARI EMPAT KALI SEMINGGU… 5<br>TIDAK PERGI … 6
E2b.	Pada waktu seperti ini, seberapa sering Anda bepergian ke Labuan Bajo, Warloka, Sape atau Bima untuk membeli makanan tidak mudah rusak seperti gula, terigu, atau beras untuk konsumsi rumah tangga Anda?		Tanggapan:<br>SEKALI SEBULAN … 1<br>DUA KALI SEBULAN … 2	SEKALI SEMINGGU … 3<br>DUA ATAU TIGA KALI SEMINGGU… 4	LEBIH DARI EMPAT KALI SEMINGGU… 5<br>TIDAK PERGI … 6

	Pembagian makanan		
E2a.	Pada waktu seperti ini, seberapa sering Anda memberikan bahan makanan ke rumah tangga lain?		Tanggapan: SERING … 1 KADANG … 2 JARANG … 3 TIDAK PERNAH … 4 ** CATATAN: Sama seperti E1
E2b.	Pada waktu seperti ini, seberapa sering Anda menerima makanan dari rumah tangga lain?		Tanggapan: SERING … 1 KADANG … 2 JARANG … 3 TIDAK PERNAH … 4

Confidential

EXP.	Modul ini adalah tentang konsumsi pangan dan minuman dan pengalaman-pengalaman ketahanan pangan Anda dan rumah tangga Anda. Modul ini akan diulangi lagi sehingga kami bisa mengetahui konsumsi dan pengalamann anda pada musim hujan dan musim kering. Responden haruslah kepala rumah tangga perempuan berusia 18 – 49 tahun.

BAGIAN 1: KONSUMSI PANGAN

EXP	Saya ingin Anda mendeskripsikan pangan (makanan dan kudapan) yang dikonsumsi seluruh anggota keluarga Anda, entah di rumah ataupun di mana saja, selama tujuh hari terakhir, yaitu dari [HARI ke HARI]; dan makanan yang Anda konsumsi pada kemarin siang dan malam, dan oleh [NAMA], anak Anda yang paling muda berusia antara 6 bulan hingga 5 tahun, pada kemarin siang dan malam. Pikirkan makanan yang betul-betul Anda makan dan bukan makanan yang pernah Anda makan atau biasa Anda makan pada musim-musim saat ini.<br>CAT : jika hari survey adalah SEN □ periode survey adalah dari SEN ke MING sebelumnya; SEL → SEL ke SEN; RAB → RAB ke SEL; KAM → KAM ke RAB; FRI → JUM ke KAM; SAB → SAB ke JUM; MING → MING ke SAB		
D	1A	Pertama, pikirkan semua anggota keluarga Anda, Apakah anggota keluarga Anda ada yang mengonsumsi makanan ini pada tujuh hari terakhir?	Tanggapan<br>YA .... 1 TIDAK ... 2
	1B	Berapa hari anggota keluarga Anda mengonsumsi makanan-makanan ini?	Jumlah hari (1 – 7)
	2	Sekarang pikirkan diri Anda sendiri, apakah Anda mengonsumsi makanan ini pada kemarin siang atau malam?	YA .... 1 TIDAK ... 2
	3	Sekarang pikirkan hanya [NAMA], anak termuda anda yang berusia 6 bulan hingga 5 tahun, apakah [NAMA] mengonsumsi makanan ini kemarin siang atau malam?	YA .... 1 TIDAK ... 2

					1A	1B	2	3
	KODE		Kategori Pangan	Deskripsi /Penjelasan	SEMUA ANGGOTA KELUARGA	JUMLAH HARI	RESPONDEN	ANAK
	FCS	DD						
	A	A	Bahan Pangan dari gandum	Nasi, beras ketan, mie, roti makanan lain yang terbuat dari gandum				
		B	Umbi putih dan umbi dan pisang tanduk	Ubi kayu, ubi outih,, kentang, atau makanan laun yang terbuat dari akar berdaging putih atau umbi atau pisang tanduk.				
		C	Kacang – kacangan (kacang merah, kacang polong, kacang lentil)	Kacang polong (segar atau kering, lentil atau produk kacang lainnya termasuk tempe/tahu				
		D	Sejenis kacang tanah dan biji-bijian	Kacang tanah, kacang mete, pasta atau olesan kacang-kacangan				
		E	Susu dan produk susu	Susu,keju, yogurt, atau produk susu lain tapi TIDAK termasuk mentega, es krim, krim atau krim asam				
		G	Daging	Daging sapi, daging babi, daging domba, daging kambing , daging kelinci, daging bebek, burung laing, atau daging hewan liar lain.				
		F	Daging Organ	Hati, ginjal, jantung atau organ lainnya, atau makan berbahan dasar darah, termasuk dari hewan liar				
		H	Semua ikan atau hewan laut	Ikan segar atau kering, termasuk ikan kaleng atau hewan laut lain (bulu babi, tatehe)				
			Ikan - Segar					
			Jika ya, jenis ikan segar apa yang Anda makan?					
			Ikan – Kering					
			Jika ya, jenis ikan kering apa yang					

Confidential

			Anda makan?					
			Ikan - Kaleng					
			Jika ya, jenis ikan kaleng apa yang Anda makan?					
			Hewan Laut					
			Jika ya, jenis hewan laut apa yang Anda makan?					
			Hewan laut lainnya					
			Jika ya, jenis hewan laut lainnya apa yang Anda makan?					
			Telur	Telur dari unggas atau burung lainnya				
			Akar, umbi atau sayuran kaya Vitamin A	Labu, wortel, labu kuning, ubi jalar yang berwarna kuning atau oranye di dalam, contoh local …				
			Sayuran berdaun hijau tua	Daun ubi, sawi, daun labu, daun ubi jalur, kangkong, bayam				
			Buah-buahan kaya Vitamin A	Manga masak, papaya masak (bukan oranye)				
			Sayuran lain	Kol, jagung, terung, bawang, rumput laut				
			Buah-buahan lain	Alpukat, pisang, jeruk, nanas, rambutan, asam, semangka, daging kelapa				
			Minyak dan lemak lain	Minyak, lemak, atau mentega yang ditambahkan pada makanan atau digunakan saat memeasak termasuk minyak yang diekstrak dari kacang, buah dan biji-bijian, dan semua lemak hewan, santan.				
			Kudapan manis	Gula, madu, selai, permen, kue, pastry, keik, dan makanan manis lain (minuman manis)				
			Kudapan gurih dan goreng-gorengan	Keripik, gorengan, atau kudapan gorengan lain				
			Bumbu dan penyedap rasa	Bahan yang digunakan dalam jumlah sedikit untuk memberi rasa, sepeti cabai, bumbu-bumbu, bawang putih, daun-daunan, bubuk ikan, saus tomat, penyedap rasa, atau biji-bijian				
			Minuman dan makanan lain	Kopi atau teh tawar, kaldu bening, alcohol, acar, zaitun dan sejenisnya, air kelapa				

BAGIAN 2: KERAGAMAN DIET ANAK-ANAK DAN BAYI

EXP:	Pertanyaan ini tentang makanan yang dikonsumsi [NAMA] kemarin siang dan malam		
D2.	Berapa banyak mkaan yang dikonsumsi [NAMA] kemarin siang dan malam?		Tanggapan:<br>Jumlah makanan

Confidential

BAGIAN 3: KERAWANAN PANGAN RUMAH TANGGA

EXP.	Sekarang Saya ingin bertanya pada Anda beberapa pertanyaan mengenai pengalaman Anda dan keluarga dalam mendapatkan pangan. Pertanyaan ini berkaitan dengan **setiap orang di rumah tangga Anda** dengan periode waktu dalam empat minggu terakhir (30 hari).					
E1.	Dalam empat minggu terakhir … <bacakan pertanyaan berikut><br>Jika jawabannya YA …<br>Seberapa sering Anda <ringkaskan pernyataan> ?<br>CATATAN: JARANG (satu atau dua kali dalam 4 minggu) KADANG (3 sampai 10 kali dalam 4 minggu SERING (lebih dari 10 kali dalam 4 minggu)		A.	Tanggapan:<br>YA … 1<br>TIDAK… 2<br>Jika TIDAK LONCAT ke berikutnya	B.	Frekuensi
	a.	Apakah Anda **khawatir** jika keluarga Anda tidak akan memiliki cukup makanan?				
	b.	Apakah Anda atau anggota keluarga yang lain **tidak mampu untuk mengonsumsi jenis makanan yang diinginkan** karena kurang sumber daya?				
	c.	Apakah Anda atau anggota keluarga yang lain harus **makan variasi makanan yang terbatas** karena kurangnya sumberdaya?				
	d.	Apakah Anda atau anggota keluarga Anda harus **makan makanan yang sebenarnya tidak ingin Anda makan** karena kurangnya sumberdaya untuk mendapatkan jenis makanan lainnya?				
	e.	Apakah Anda atau anggota keluarga Anda harus **makan makanan lebih sedikit** dari yang Anda pikir Anda butuhkan karena kurangnya makanan?				
	f.	Apakah Anda atau anggota keluarga yang lain harus **makan makanan lebih sedikit** dalam satu hari karena kurangnya makanan?				
	g.	Pernahkan **tidak ada makanan apapun untuk dimakan** di keluarga anda karena kurangnya sumberdaya untuk mendapatkan makanan?				
	h.	Apakah Anda atau anggota keluarga yang lain **pergi tidur malam dalam keadaan lapar** karena tidak ada cukup makanan?				
	i.	Apakah Anda atau anggota keluarga yang lain **beraktifitas seharian atau semalaman tanpa makan** karena tidak ada makanan yang cukup?				
	Tanggapan:<br>E1.B. JARANG … 1 KADANG … 2 SERING … 3					

	Perolehan makanan				
E2a.	Pada waktu seperti ini, seberapa sering Anda bepergian ke Labuan Bajo, Warloka, Sape atau Bima untuk membeli makanan segar seperti buah dan sayur untuk konsumsi rumah tangga Anda?		Tanggapan:<br>SEKALI SEBULAN … 1<br>DUA KALI SEBULAN … 2	SEKALI SEMINGGU … 3<br>DUA ATAU TIGA KALI SEMINGGU… 4	LEBIH DARI EMPAT KALI SEMINGGU… 5<br>TIDAK PERGI … 6
E2b.	Pada waktu seperti ini, seberapa sering Anda bepergian ke Labuan Bajo, Warloka, Sape atau Bima untuk membeli makanan tidak mudah rusak seperti gula, terigu, atau beras untuk konsumsi rumah tangga Anda?		Tanggapan:<br>SEKALI SEBULAN … 1<br>DUA KALI SEBULAN … 2	SEKALI SEMINGGU … 3<br>DUA ATAU TIGA KALI SEMINGGU… 4	LEBIH DARI EMPAT KALI SEMINGGU… 5<br>TIDAK PERGI … 6

	Pembagian makanan		
E2a.	Pada waktu seperti ini, seberapa sering Anda memberikan bahan makanan ke rumah tangga lain?		Tanggapan: SERING … 1 KADANG … 2 JARANG … 3 TIDAK PERNAH … 4 ** CATATAN: Sama seperti E1
E2b.	Pada waktu seperti ini, seberapa sering Anda menerima makanan dari rumah tangga lain?		Tanggapan: SERING … 1 KADANG … 2 JARANG … 3 TIDAK PERNAH … 4

Confidential

MODUL C: PENGAMBILAN KEPUTUSAN, KEPEMILIKAN ASET, KEANGGOTAAN, SIKAP DAN PENGETAHUAN

EXP.	Rangkaian pertanyaan berikut ini adalah pengaturan rumah tangga anda dan sikap terhadap beberapa hal.<br>Modul ini untuk diberikan pada responden **kepala rumah tangga perempuan** (responden dari modul lainnya) dan pasangan **kepala rumah tangga laki-laki.**

MODUL C: PENGAMBILAN KEPUTUSAN, KEPEMILIKAN ASET, KEANGGOTAAN, SIKAP DAN PENGETAHUAN

EXP.	Rangkaian pertanyaan berikut ini adalah pengaturan rumah tangga anda, partisipasi dalam aktifitas komuniast, sikap dan pengetahuan Anda tentang beberapa hal			
F1	Dalam pertanyaan-pertanyaan berikut ini, beri peringkat keterlibatan Anda dalam pengambilan keputusan pada area yang ditentukan dari skala 0 – 10.<br>Jika Anda tidak memiliki keterlibatan sama sekali dalam keputusan dalam beberapa area, Anda akan memilih 0, jika Anda sendiri satu-satunya yang memutuskan maka Anda akan memilih 10, tapi jika Anda membagi keputusan secara sama rata dengan pasangan Anda atau orang lain (seperti orangtua atau mertua) Anda akan memilih 5.			
	Apakah Anda pernah terlibat dalam menentukan :			Tanggapan:<br>0-10<br><br>0 – tidak terlibat dalam pengambilan keputusan<br>... - sesekali terlibat<br>5 - keterllibatan sama rata<br>.... – terlibat dalam hampir semua putusan<br>10 - selalu membuat putusan sendiri
	a	Aktifitas menangkap ikan, termasuk kapan, dimana dan bagaimana		
	b	Aktifitas produktif lain, seperti memulai atau menjalankan kios atau bisnis kecil		
	c	Memelihara hewn ternak (seperti ayam, bebek, kambing) untuk makan		
	d	Jenis dan jumlah makanan untuk keluarga Anda		
	e	Memiliki sumber pendapatan sendiri		
	f	Manajemen keuangan keluarga (pendapatan dari suami dan istri)		
	g	Kesehatan Anda sendiri termasuk pergi ke Pustu		
	h	Keputusan untuk melanjutkan pendidikan Anda		
	I	Keputusan untuk menggunakan kontrasepsi dalam keluarga berencana		
	j	Pasangan perempuan mengunjungi pasar di Labuan Bajo		
F2	Saya ingin mengetahui pendapat Anda tentang mengatur rumahtangga dan aktifitas sehari-hari			
	Bisakah Anda memeberitahu saya apakah Anda setuju atau tidak setuju dengan pernyataan berikut?			
	a	Tindakan saya hanya sebagian sebab saya akan terkena masalah dengan seseorang jika saya bertingkah berbeda		Tanggapan:<br>Sepenuhnya setuju ... 1<br>Agak setuju .... 2<br>Sepenuhnya tidak setuju ... 3<br>TT ... 4
	b	Saya berbuat sesuatu agar orang tidak berpikir buruk tentang saya		
	c	Saya berbuat sesuatu sebab saya secara pribadi berpikir itu adalah hal baik untuk dilakukan		

F3	Dalam rangkaian pertanyaan berikut ini, tolong beritahukan apakah Anda setuju atau tidak setuju dengan pernyataan berikut.<br>Kami tertarik pada pendapat pribadi Anda, dan Anda tidak perlu memberitahu kami apabila Ada memiliki pengalaman pribadi mengenai situasi yang digambarkan						
	a.	Dalam pendapat Anda, seoranng suami dibenarkan memukul atau menghajar istrinya dalam situasi berikut:					
		A	B	C	D	E	Tanggapan:<br>Setuju ... 1<br>Tidak Setuju ... 2<br>Tidak Tahu ... 3
		Keluar tanpa memberitahukan suami	Menghiraukan anak-anak	Beradu mulut dengannya	Menolak berhubungan seksual	Menghanguskan makanan	
	b	Dalam pendapat Anda, seoranng suami dibenarkan menghukum secara verbal istrinya (seperti meneriakinnya atau memanggil dengan nama yang menghina) dalam situasi berikut:					
		A	B	C	D	E	Tanggapan:<br>Setuju ... 1<br>Tidak Setuju ... 2<br>Tidak Tahu ... 3
		Keluar tanpa memberitahukan suami	Menghiraukan anak-anak	Beradu mulut dengannya	Menolak berhubungan seksual	Menghanguskan makanan	

F4	Pertanyaan berikut adalah tentang keterlibatan dalam aktifitas masyarakat dan manajemen waktu Anda			
	a	Apakah Anda adalah anggota dari kelompok apapun di komunitas Anda?<br>*Contoh: produsen perikanan, atau kelompok pasar, air, mikrofinanasial, keuntungan Bersama atau kelompok asuransi, asosiasi perdaganagn dan bisnis, kelompok sipil/amal, pemerintah local, kelompok agama, kelompok lainnya.*		Tanggapan:<br>YA .... 1 TIDAK ... 2<br>Jika YA, sebutkan nama/jenis kelompok.
	b.	Apakah Anda merasa nyaman berbicara di depan umum untuk membantu memutuskan infastuktur (seperti sediaan air, atau tanggul) yang akan dibangun di komunitas Anda?		Tanggapan:<br>Tidak, sama sekali tidak nyaman ... 1<br>Ya, dengan beberapa kesulitan ... 2<br>Ya, nyaman ... 3<br>Tidak Tahu ... 4

Confidential

	c	*Pertanyaan berikut adalah tentang seberapa puas Anda dengan waktu yang Anda miliki untuk melakukan hal-hal yang Anda nikmati. Tolong berikan pendapat Anda pada skal 1 hingga 10, dimana 1 berarti Anda tidak puas dan 10 berarti Anda sangat puas. Jikatidak keduanya, berarti di tengah0tengah, atau 5 pada skala tersebut.*<br>Seberapa puas Anda terhadap waktu yang ada untuk Ada untuk bersantai seperti mengunjungi keluarga, menonton televisi atau berolahraga?		Tanggapan:<br>1 – 10<br><br>0 – tidak puas<br>10 – sangat puas
F5	Pertanyaan berikut adalah tentang pangan yang keluarga Anda makan dan kesehatan keluarga Anda			
	a	Apa saja yang Anda lakukan agar tetap sehat?<br>*Tanyakan lebih lanjut mengenai tanggapan tambahan (ada lagi?)*		Pilihan Tanggapan:<br>Makan makanan yang beragam (5 kelompok makanan)<br>Makan banyak buah dan sayuran<br>Makan protein (ikan, susu, telur)<br>Membatasi asupan makanan manis, asin dan berlemak<br>Sarapan<br>Banyak minum air putih<br>Mencuci tangan dengan sabun dan air mengalir<br>Beraktifitas fisik yang cukup<br>Mempertahankan berat badan<br>Tidak merokok<br>Lainnya (sebutkan)
	b	Apa saya yang wanita hamil dan menyusui lalukan agar bisa tetap sehat?<br>*Tanyakan lebih lanjut mengenai tanggapan tambahan (ada lagi?)*		Pilihan Tanggapan:<br>Makan beragam makanan<br>Makan makanan kaya protein, zat besi dan kalsium<br>Minum asam folat (atau makanan yang mengandung)<br>Membatasi makanan dengan kadar garam tinggi<br>Membatasi konsumsi kafein<br>Lainnya (sebutkan)
	c	Saat-saat penting yang mana yang Anda harus mencuci tangan?<br>*Tanyakan lebih lanjut mengenai tanggapan tambahan (ada lagi?)*		Pilihan Tanggapan:<br>Setelah pergi ke toilet … 1<br>Setelah membersihkan bayi/mengganti popok bayi … 2<br>Sebelum menyediakan atau mengurus makanan … 3<br>Sebelum memeberi makan anak/makan … 4<br>Setelah mengurus makanan mentah …. 5<br>Setelah mengurus sampah … 6<br>Lainnya (sebutkan)
	d	Menggunakan sabun untuk mencuci tangan adalah tidak penting		Tanggapan:<br>BENAR … 1 SALAH … 2

Confidential

FORMULIR PENJELASAN DAN PERSETUJUAN

RESPONDENT 1 – LAKI LAKI

<INSERT INFORMED CONTEST TEXT>

MODUL C: PENGAMBILAN KEPUTUSAN, KEPEMILIKAN ASET, KEANGGOTAAN, SIKAP DAN PENGETAHUAN

EXP.	Rangkaian pertanyaan berikut ini adalah pengaturan rumah tangga anda dan sikap terhadap beberapa hal.<br>Modul ini untuk diberikan pada responden **kepala rumah tangga perempuan** (responden dari modul lainnya) dan pasangan **kepala rumah tangga laki-laki.**

MODUL C: PENGAMBILAN KEPUTUSAN, KEPEMILIKAN ASET, KEANGGOTAAN, SIKAP DAN PENGETAHUAN

EXP.	Rangkaian pertanyaan berikut ini adalah pengaturan rumah tangga anda, partisipasi dalam aktifitas komuniast, sikap dan pengetahuan Anda tentang beberapa hal			
F1	Dalam pertanyaan-pertanyaan berikut ini, beri peringkat keterlibatan Anda dalam pengambilan keputusan pada area yang ditentukan dari skala 0 – 10.<br>Jika Anda tidak memiliki keterlibatan sama sekali dalam keputusan dalam beberapa area, Anda akan memilih 0, jika Anda sendiri satu-satunya yang memutuskan maka Anda akan memilih 10, tapi jika Anda membagi keputusan secara sama rata dengan pasangan Anda atau orang lain (seperti orangtua atau mertua) Anda akan memilih 5.			
	Apakah Anda pernah terlibat dalam menentukan :			Tanggapan:<br>0-10<br><br>0 – tidak terlibat dalam pengambilan keputusan<br>… - sesekali terlibat<br>5 - keterllibatan sama rata<br>…. – terlibat dalam hampir semua putusan<br>10 - selalu membuat putusan sendiri
	a	Aktifitas menangkap ikan, termasuk kapan, dimana dan bagaimana		
	b	Aktifitas produktif lain, seperti memulai atau menjalankan kios atau bisnis kecil		
	c	Memelihara hewn ternak (seperti ayam, bebek, kambing) untuk makan		
	d	Jenis dan jumlah makanan untuk keluarga Anda		
	e	Memiliki sumber pendapatan sendiri		
	f	Manajemen keuangan keluarga (pendapatan dari suami dan istri)		
	g	Kesehatan Anda sendiri termasuk pergi ke Pustu		
	h	Keputusan untuk melanjutkan pendidikan Anda		
	I	Keputusan untuk menggunakan kontrasepsi dalam keluarga berencana		
	j	Pasangan perempuan mengunjungi pasar di Labuan Bajo		
F2	Saya ingin mengetahui pendapat Anda tentang mengatur rumahtangga dan aktifitas sehari-hari			
	Bisakah Anda memeberitahu saya apakah Anda setuju atau tidak setuju dengan pernyataan berikut?			
	a	Tindakan saya hanya sebagian sebab saya akan terkena masalah dengan seseorang jika saya bertingkah berbeda		Tanggapan:<br>Sepenuhnya setuju … 1<br>Agak setuju …. 2<br>Sepenuhnya tidak setuju … 3<br>TT … 4
	b	Saya berbuat sesuatu agar orang tidak berpikir buruk tentang saya		
	c	Saya berbuat sesuatu sebab saya secara pribadi berpikir itu adalah hal baik untuk dilakukan		

F3	Dalam rangkaian pertanyaan berikut ini, tolong beritahukan apakah Anda setuju atau tidak setuju dengan pernyataan berikut.<br>Kami tertarik pada pendapat pribadi Anda, dan Anda tidak perlu memberitahu kami apabila Ada memiliki pengalaman pribadi mengenai situasi yang digambarkan							
	a.	Dalam pendapat Anda, seoranng suami dibenarkan memukul atau menghajar istrinya dalam situasi berikut:						
		A	B	C	D	E	Tanggapan:<br>Setuju … 1<br>Tidak Setuju … 2<br>Tidak Tahu … 3	
		Keluar tanpa memberitahukan suami	Menghiraukan anak-anak	Beradu mulut dengannya	Menolak berhubungan seksual	Menghanguskan makanan		
	b	Dalam pendapat Anda, seoranng suami dibenarkan menghukum secara verbal istrinya (seperti meneriakinnya atau memanggil dengan nama yang menghina) dalam situasi berikut:						
		A	B	C	D	E	Tanggapan:<br>Setuju … 1<br>Tidak Setuju … 2<br>Tidak Tahu … 3	
		Keluar tanpa memberitahukan suami	Menghiraukan anak-anak	Beradu mulut dengannya	Menolak berhubungan seksual	Menghanguskan makanan		
F4	Pertanyaan berikut adalah tentang keterlibatan dalam aktifitas masyarakat dan manajemen waktu Anda							
	a	Apakah Anda adalah anggota dari kelompok apapun di komunitas Anda?		Tanggapan:				

Confidential

		Contoh: produsen perikanan, atau kelompok pasar, air, mikrofinanasial, keuntungan Bersama atau kelompok asuransi, asosiasi perdaganagn dan bisnis, kelompok sipil/amal, pemerintah local, kelompok agama, kelompok lainnya.		YA .... 1 TIDAK ... 2<br>Jika YA, sebutkan nama/jenis kelompok.
	b.	Apakah Anda merasa nyaman berbicara di depan umum untuk membantu memutuskan infastuktur (seperti sediaan air, atau tanggul) yang akan dibangun di komunitas Anda?		Tanggapan:<br>Tidak, sama sekali tidak nyaman ... 1<br>Ya, dengan beberapa kesulitan ... 2<br>Ya, nyaman ... 3<br>Tidak Tahu ... 4
	c	*Pertanyaan berikut adalah tentang seberapa puas Anda dengan waktu yang Anda miliki untuk melakukan hal-hal yang Anda nikmati. Tolong berikan pendapat Anda pada skal 1 hingga 10, dimana 1 berarti Anda tidak puas dan 10 berarti Anda sangat puas. Jikatidak keduanya, berarti di tengah0tengah, atau 5 pada skala tersebut.*<br>Seberapa puas Anda terhadap waktu yang ada untuk Ada untuk bersantai seperti mengunjungi keluarga, menonton televisi atau berolahraga?		Tanggapan:<br>1 – 10<br><br>0 – tidak puas<br>10 – sangat puas
F5	Pertanyaan berikut adalah tentang pangan yang keluarga Anda makan dan kesehatan keluarga Anda			
	a	Apa saja yang Anda lakukan agar tetap sehat?<br>*Tanyakan lebih lanjut mengenai tanggapan tambahan (ada lagi?)*		Pilihan Tanggapan:<br>Makan makanan yang beragam (5 kelompok makanan)<br>Makan banyak buah dan sayuran<br>Makan protein (ikan, susu, telur)<br>Membatasi asupan makanan manis, asin dan berlemak<br>Sarapan<br>Banyak minum air putih<br>Mencuci tangan dengan sabun dan air mengalir<br>Beraktifitas fisik yang cukup<br>Mempertahankan berat badan<br>Tidak merokok<br>Lainnya (sebutkan)
	b	Apa saya yang wanita hamil dan menyusui lalukan agar bisa tetap sehat?<br>*Tanyakan lebih lanjut mengenai tanggapan tambahan (ada lagi?)*		Pilihan Tanggapan:<br>Makan beragam makanan<br>Makan makanan kaya protein, zat besi dan kalsium<br>Minum asam folat (atau makanan yang mengandung)<br>Membatasi makanan dengan kadar garam tinggi<br>Membatasi konsumsi kafein<br>Lainnya (sebutkan)
	c	Saat-saat penting yang mana yang Anda harus mencuci tangan?<br>*Tanyakan lebih lanjut mengenai tanggapan tambahan (ada lagi?)*		Pilihan Tanggapan:<br>Setelah pergi ke toilet ... 1<br>Setelah membersihkan bayi/mengganti popok bayi ... 2<br>Sebelum menyediakan atau mengurus makanan ... 3<br>Sebelum memeberi makan anak/makan ... 4<br>Setelah mengurus makanan mentah .... 5<br>Setelah mengurus sampah ... 6<br>Lainnya (sebutkan)
	d	Menggunakan sabun untuk mencuci tangan adalah tidak penting		Tanggapan:<br>BENAR ... 1 SALAH ... 2

Confidential

A8a. ** terjemahkan ke Bahasa Indonesia dan Bahasa Sama

Memancing itu pekerjaan yang sulit.	
Anak-anak belajar dengan giat di sekolah.	
Anak itu sedang membaca buku.	
Orangtua mencintai anak mereka.	

A8b.

17 + 6 = ______

5,000 x 8 = _______

Pengukuran Tinggi dan Berat Badan

Questionnaire code: ____________________

Date: _____________________

Silakan bawa kartu ini ___________ agar tinggi dan berat badan Anda dan anak paling muda Anda yang berusia 6 bulan hingga 5 tahun diukur. Tolong diisi pada: ________________________

Person	Age (Y/M)	Gender (L/P)	Weight (kg)	Method (TS/N)	Height (cm)	Method (L/S)	Recorded by:
Mother		P					
Child 1							

Terima kasih.

Confidential
